# Shorter life and reduced fecundity can increase colony fitness in virtual *Caenorhabditis elegans*

**DOI:** 10.1111/acel.13141

**Published:** 2020-04-16

**Authors:** Evgeniy R. Galimov, David Gems

**Affiliations:** ^1^ Institute of Healthy Ageing, and Research Department of Genetics, Evolution and Environment University College London London UK

**Keywords:** adaptive death, *C. elegans* ecology, evolution of aging, evolutionary modelling, inclusive fitness

## Abstract

In the nematode *Caenorhabditis elegans*, loss of function of many genes leads to increases in lifespan, sometimes of a very large magnitude. Could this reflect the occurrence of programmed death that, like apoptosis of cells, promotes fitness? The notion that programmed death evolves as a mechanism to remove worn out, old individuals in order to increase food availability for kin is not supported by classic evolutionary theory for most species. However, it may apply in organisms with colonies of closely related individuals such as *C. elegans* in which largely clonal populations subsist on spatially limited food patches. Here, we ask whether food competition between nonreproductive adults and their clonal progeny could favor programmed death by using an in silico model of *C. elegans*. Colony fitness was estimated as yield of dauer larva propagules from a limited food patch. Simulations showed that not only shorter lifespan but also shorter reproductive span and reduced adult feeding rate can increase colony fitness, potentially by reducing futile food consumption. Early adult death was particularly beneficial when adult food consumption rate was high. These results imply that programmed, adaptive death could promote colony fitness in *C. elegans* through a consumer sacrifice mechanism. Thus, *C. elegans* lifespan may be limited not by aging in the usual sense but rather by apoptosis‐like programmed death.

## Graphical abstract

*Caenorhabditis* *elegans* fitness can be viewed at the level of individual animals or of viscous, clonal populations of worms (or colonies). Behavior of an in silico model of *C. elegans* predicts that early death of postreproductive adults can increase colony fitness (measured as yield of dauer propagules) by reducing futile food consumption. This supports the occurrence of apoptosis‐like programmed organismal death in *C. elegans*, of the consumer sacrifice type, which limits lifespan.

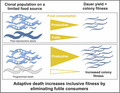

## 1 INTRODUCTION

**Figure 1:**
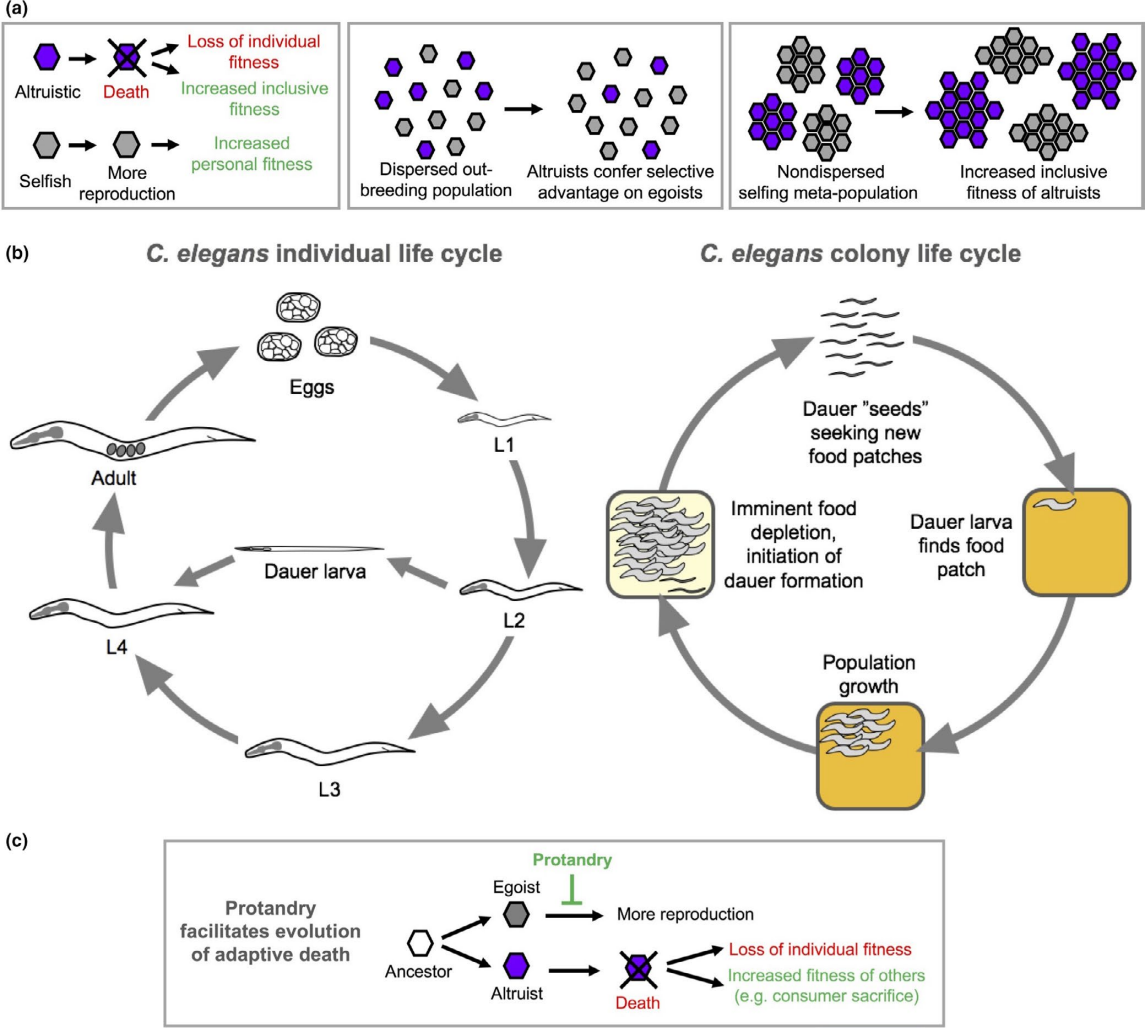
Evolution of consumer sacrifice‐type adaptive death in *Caenorhabditis elegans*. (a) General conditions for the evolution of altruistic adaptive death, for example involving consumer sacrifice (programmed death to increase food availability for kin; Lohr et al., 2019). Altruistic decrease in individual fitness can increase inclusive fitness (i.e., individual fitness + fitness of kin). In dispersed outbreeding, populations egoists outcompete altruists, but in nondispersed selfing meta‐populations altruists are fitter (note that selfish is synonymous with egoistic). (b) *C. elegans* reproductive fitness is manifested via life cycles at two levels, that of the individual nematode and that of the nematode colony. In the wild, *C. elegans* populations experience cycles of boom and bust. When a dauer larva dispersal form encounters new food patch (e.g., a rotting plant stem), it forms a colony of isogenic nematodes. When the food source is exhausted, dauer propagules disperse to found new colonies. (c) Protandrous hermaphroditism decreases individual fitness by curtailing reproduction and, theoretically, could facilitate the evolution of consumer sacrifice adaptive death by removing the possibility of selection benefits to individual nematode fitness of continuation of reproduction (i.e., egoistic cheating; Lohr et al., 2019). (a, c) Arrows indicate change, progression or consequence; green T bar indicates inhibition

Aging (senescence) is a ubiquitous feature of living organisms but does it have any biological function? Several contemporaries of Charles Darwin proposed that it does: according to August Weismann and Alfred Russel Wallace, aging serves to remove worn out individuals, thereby increasing food availability for the benefit of the species (Rose, 1991). But modern evolutionary theory, supported by population genetic analysis, indicates that extreme altruism of this type will not be favored by natural selection, at least not in organisms that exist as dispersed, outbred populations (Hamilton, 1964; Maynard Smith, 1976; Williams, 1966). This is because of the vulnerability of suicidal altruists to exploitation by nonsuicidal, nonaltruists (egoists; Figure 1a). However, this deduction does not hold for species that exist as viscous (i.e., nondispersed) populations of largely clonal individuals (Figure 1a), such as colonies of budding yeast (*Saccharomyces cerevisiae*), or populations of the nematode *Caenorhabditis elegans* on food patches (Galimov, Lohr, & Gems, 2019; Lohr, Galimov, & Gems, 2019).

*Caenorhabditis elegans* has been extensively studied as a genetic model organism to try to discover the causes of senescence, a process that is now the largest cause of human disease worldwide (including cardiovascular disease, chronic obstructive pulmonary disease, dementia, cancer and many others; Niccoli & Partridge, 2012). Numerous genes and pathways have been discovered in *C. elegans* where mutation leads to increases in lifespan (Gems & Partridge, 2013; Kenyon, 2010), sometimes of a very large magnitude (Arantes‐Oliveira, Berman, & Kenyon, 2003; Ayyadevara, Alla, Thaden, & Shmookler Reis, 2008; Houthoofd, Braeckman, & Vanfleteren, 2004), implying the presence of wild‐type mechanisms that greatly increase late‐life mortality. We recently argued that in *C. elegans*, in contrast to most metazoans (including humans), natural selection could have favored mechanisms that cause senescence because they shorten life. Specifically, death of postreproductive adults in viscous populations could benefit isogenic larvae and reproductive adults by increasing food availability, an example of consumer sacrifice adaptive death (Lohr et al., 2019).

Recent advances in *C. elegans* ecology suggest that its life cycle occurs at two distinct levels of organization. The lower level involves the life cycle of the individual nematode, in which fitness is measurable as the number of offspring per worm (Figure 1b). However, in the wild, *C. elegans* are found as colony‐like, clonal populations existing upon spatially restricted food patches (e.g., rotting plant stems or fruit; Schulenburg & Félix, 2017). In their natural setting, *C. elegans* exhibit a boom‐and‐bust type life cycle (Frezal & Felix, 2015). They encounter and colonize a food patch, which then leads to rapid population growth and depletion of food. Dwindling food and high population density triggers larval entry into a diapausal dispersal stage: the dauer larva (Cassada & Russell, 1975). Dauers are resistant to environmental stress and can survive without food for up to 75–80 days (Banfield, Gomez, Lee, Clarke, & Larsen, 2008; Hu, 2007; Klass & Hirsh, 1976). Dauer dispersal is aided by various animal vectors, including isopods (e.g., woodlice), snails and slugs (Schulenburg & Félix, 2017). When a dauer encounters a new food patch, it resumes development and the boom‐and‐bust cycle rolls on. At this upper level, the life cycle can be considered as that of the colony (Cutter, 2015; Lohr et al., 2019; Yin & Haag, 2019; Figure 1b). Our working hypothesis is that adaptive death promotes colony level fitness (or, from another perspective, inclusive fitness; Kramer & Meunier, 2016; Wilson & Wilson, 2008) but not individual fitness (Figure 1b); similarly, programmed death of individual cells in metazoans reduces fitness at the cell level but may increase fitness at the organismal level.

An additional feature of *C. elegans* life history that is predicted to facilitate the evolution of altruistic, adaptive death is protandrous hermaphroditism. The germline of the *C. elegans* hermaphrodite exhibits protandry, generating first sperm and then oocytes. Sperm are stored in spermathecae and used to fertilize oocytes. When sperm are depleted, reproduction ceases. Reproduction can continue for a longer period if hermaphrodites mate with males (Hodgkin & Doniach, 1997; Hughes, Evason, Xiong, & Kornfeld, 2007); however, males are extremely rare in the wild (Schulenburg & Félix, 2017). The timing of the switch from sperm to oocyte production appears to be optimized to maximize population growth rate, balancing earlier onset of fertilization against greater sperm/progeny number (Hodgkin & Barnes, 1991). Consequently, in selfing populations *C. elegans* reproductive span is a mere 2–3 days. Importantly, the short reproductive span resulting from protandry is predicted to facilitate selection for altruistic death by precluding invasion of populations by nonaltruistic egoists, since they would possess no reproductive benefit (Lohr et al., 2019; Figure 1c). Androdioecy (having hermaphrodites and males) in *C. elegans* arose by evolution from an ancestor that was gonochoristic (having females and males; Ellis & Lin, 2014; Kiontke et al., 2011; Stewart & Phillips, 2002). This led us previously to suggest that adaptive death in *Caenorhabditis* species might evolve only after the appearance of hermaphroditism (Lohr et al., 2019).

But is adaptive death in *C. elegans* a reality? Hypotheses about how particular traits might promote fitness are easy to formulate but can be difficult to prove or disprove (Williams, 1966). One way to test further for the plausibility of adaptive death is computer modelling. Previous patch occupancy model studies, including the ground‐breaking work by J. M. J. Travis, have shown that in viscous populations, shorter lifespan can increase fitness (Dytham & Travis, 2006; Markov, 2012; Travis, 2004). In one such model that included resource consumption by adults, lifespan was found to be inversely correlated with food consumption rate (Werfel, Ingber, & Bar‐Yam, 2015, 2017), consistent with consumer sacrifice. Here, we describe a new computer model recapitulating key features of *C. elegans* life history; this contrasts with previous models where simulations used relatively abstract organism representations. We describe how the behavior of the model supports the view that programmed death in *C. elegans* could increase colony level fitness through a consumer sacrifice mechanism and that protandrous hermaphroditism could facilitate selection for adaptive death. Behavior of the model also revealed other, unexpected mechanisms by which reduction of individual fitness could enhance colony fitness by minimizing food consumption that is futile (i.e., does not lead to increased dauer production).

## 2 RESULTS

### 2.1 Design of the in silico *C. elegans* colony life cycle model

**Figure 2:**
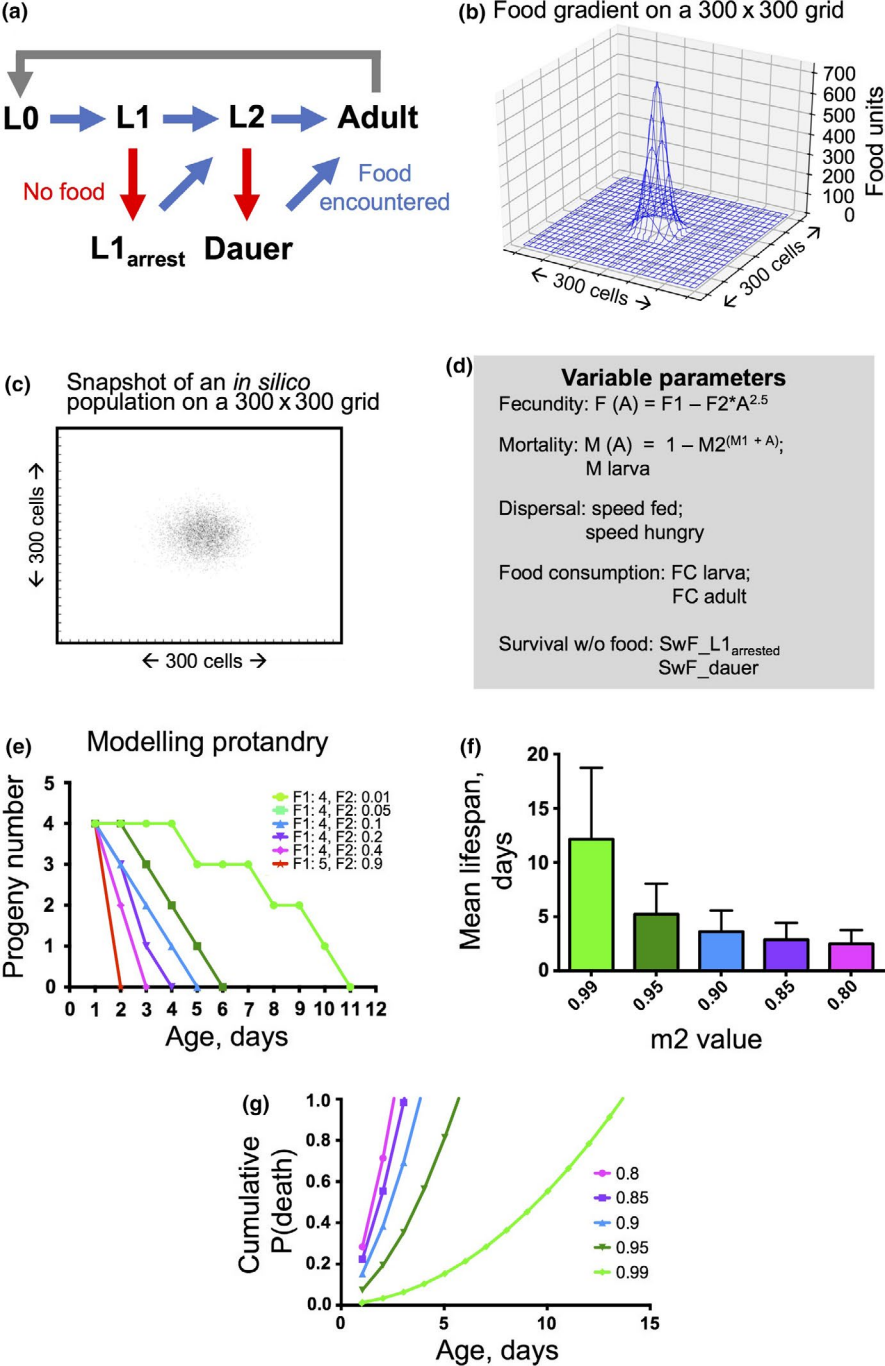
Design of the in silico *Caenorhabditis elegans* model. (a) *C. elegans* stages included in the model: early stages are reduced to L0 (egg), L1 and L2. In the absence of food, L1 and L2 stages can undergo developmental arrest (L2s becoming dauers) and survive a set number of days; see (d). Where arrested individuals encounter food, they resume development into L2s or adults, respectively. (b) For modelling, we use a 300 × 300 grid where food is distributed as a 2D Gaussian in the centre of the grid. (c) Snapshot of a growing virtual worm population on a virtual food patch. Only dauers are shown here for timepoint 15. The parameters are: no reproductive decline, adult food consumption—1,000, M2 = 0.99, dispersal speed—9, progeny production rate—50 progeny per timepoint. (d) Eleven parameters that determine the model. F1 and F2 define reproductive schedule and reproductive rate. M_larva, M1 and M2 set larval mortality and adult mortality as a function of age. Other parameters control dispersal speed, food consumption rate and survival without food for arrested larvae. (e) Illustration of how parameters F1 and F2 determine selected reproductive schedules used in simulations. (f) Estimated mean lifespans for M1 = 0.5 and different M2 values (means of 5,000 trials). (g) Cumulative probability of death is where M1 = 0.5 and with different values of M2

An in silico model organism was designed that resembles *C. elegans*. In the *C. elegans* life cycle adulthood is preceded by four larval stages, L1–L4 (Wood, 1988; Figure 1b). If eggs hatch in the absence of food, L1s can form a limited dispersal stage, which can survive for several weeks (Baugh, 2013; Johnson, Mitchell, Kline, Kemal, & Foy, 1984). With low food and high population density, as populations transition from boom to bust, L1 larvae can also develop via the L2d stage to the long‐lived dauer dispersal stage (Hu, 2007; Klass & Hirsh, 1976). In our simplified *C. elegans* model, we included only L1, L1_arrested_ (in the absence of food), L2, dauer and adult (Figure 2a).

To model a single boom‐and‐bust cycle, we created a dwelling space with a grid of 300 × 300 cells. In the centre of the grid, a food patch was placed which can be represented as a 2D Gaussian such that the food level maximum was at the centre of the grid (Figure 2b). The starting amount of food (in most cases) was ~10^6^ food units. The simulations were started by placing (in most cases) a single larva in the food patch and then running the model to observe population growth (Figure 2c) and dauer production under different sets of life history parameters. In the absence of food, due to either food depletion or worm exit from the food patch, L1s became L1_arrested_ and L2s became dauers.

Variables specifying five life history traits were defined in each version of virtual worm to assess effects on fitness: adult reproductive schedule, mortality rate, migration speed, survival without food (in days) and food consumption rate. Fecundity and mortality rate were set as a function of increasing age (Figure 2d). The fecundity of larvae was 0, and for adults, age‐specific progeny production was determined by the formula Number of progeny (age) = F1 − F2*age^2.5^, where F1 is the number of progeny produced on day 1, and F2 is the parameter determining the rate at which fertility declines with age (Figure 2e). Where F2 = 0, there is no reproductive decline with age. Mortality was set as a probability of death, that is at each timepoint (day) for each individual the coin is tossed with a set probability of dying. Larval mortality was set at 0.05 per day for all larval stages, except dauers for which it was 0.005 per day. For adults the probability of death was determined by the formula *p*_death_ (age) = 1 − M2^(M1 + age)^. Here, M2 determines the speed at which *p*_death_ (probability of dying) approaches 1. Where M2 = 1, aging does not occur. In our model, we varied M2 from 0.99 (mean lifespan 12 days) to 0.8 (mean lifespan 2.4 days), see Figure 2f,g. Although under standard laboratory conditions lifespan is 2–3 weeks (Klass, 1977), *C. elegans* in the wild certainly experience much shorter lifespans to which genetic determinants are likely to contribute (Lohr et al., 2019); thus, though our model tests benefits of programmed aging, we assume that extrinsic factors (especially infection and starvation) will amplify its effects. Available evidence suggests that wild *C. elegans* colonies typically exist as clonal populations of selfing hermaphrodites with barely any males (Barriere & Felix, 2007; Frezal & Felix, 2015; Petersen et al., 2015; Richaud, Zhang, Lee, Lee, & Félix, 2018). Therefore, our in silico *C. elegans* correspond to hermaphrodite‐only populations, where all progeny contain the same genes as their parent.

Because we were interested in inclusive fitness (rather than individual fitness), we measured dauer yield per colony rather than number of progeny per worm (Figure 1b). Colony dauer yield is a metric of the capacity to disperse and found new colonies, that is of colonies to replicate. This involves an implicit assumption that colony dauer yield is directly proportional (1:1) with the capacity to found new colonies, but in the real world more complex relationships may exist.

Animals were assigned two locomotory modes: that when hungry and that when fed. The speed for hungry worms was set at maximal (9 cells/day). This mimicked the behavior of *C. elegans*, which congregate and show reduced motility within food patches (Gray et al., 2004; Sawin, Ranganathan, & Horvitz, 2000). For fed worms, speed was varied from 1 to 9 cells/day. We also defined survival times for larvae in the absence of food: 6 days for L1s and 30 days for dauers, consistent with the longer dauer survival time (Johnson et al., 1984; Klass & Hirsh, 1976). For simplicity's sake, our model did not include an effect of food deprivation on adult survival. Finally, we defined two food consumption parameters; for most conditions they were for adults 1,000 units per cycle (or 50–1,000, when varied) and for larvae 50 units per cycle (or 25–500 when varied; Figure 2d). Figure S1 shows how the model parameters determine the relationships between quantities of food, adults and progeny, to aid the reader in interpretation of the results of simulations (see below).

Simulations were carried out to test whether there are life history parameter combinations (or model *genotypes*) where earlier death can promote fitness and, if there are, to investigate how life history parameters interact to make death beneficial. For each genotype, we performed 100 repeat simulations and calculated mean output values. For further details of the model, see Section 4.

We first asked whether shorter lifespan could increase fitness in our *C. elegans*‐like in silico model. In previous patch occupancy‐based models, shorter lifespan could sometimes evolve when fertility declined with age, and progeny dispersal was limited (Markov, 2012; Travis, 2004). We tested five aging rates that gave mean lifespans ranging from 2.4 to 12 days and nine dispersal speeds, ranging from 1 to 9 cells/day. Simulations were run to estimate fitness for all 45 aging rate × dispersal speed combinations. Dauers were set to survive without food for 30 days, and larval food consumption was 50 food units/day (i.e., 20 times lower than adults).

### 2.2 The effect of lifespan on fitness depends on the progeny production rate when there is no age‐related reproductive decline

**Figure 3:**
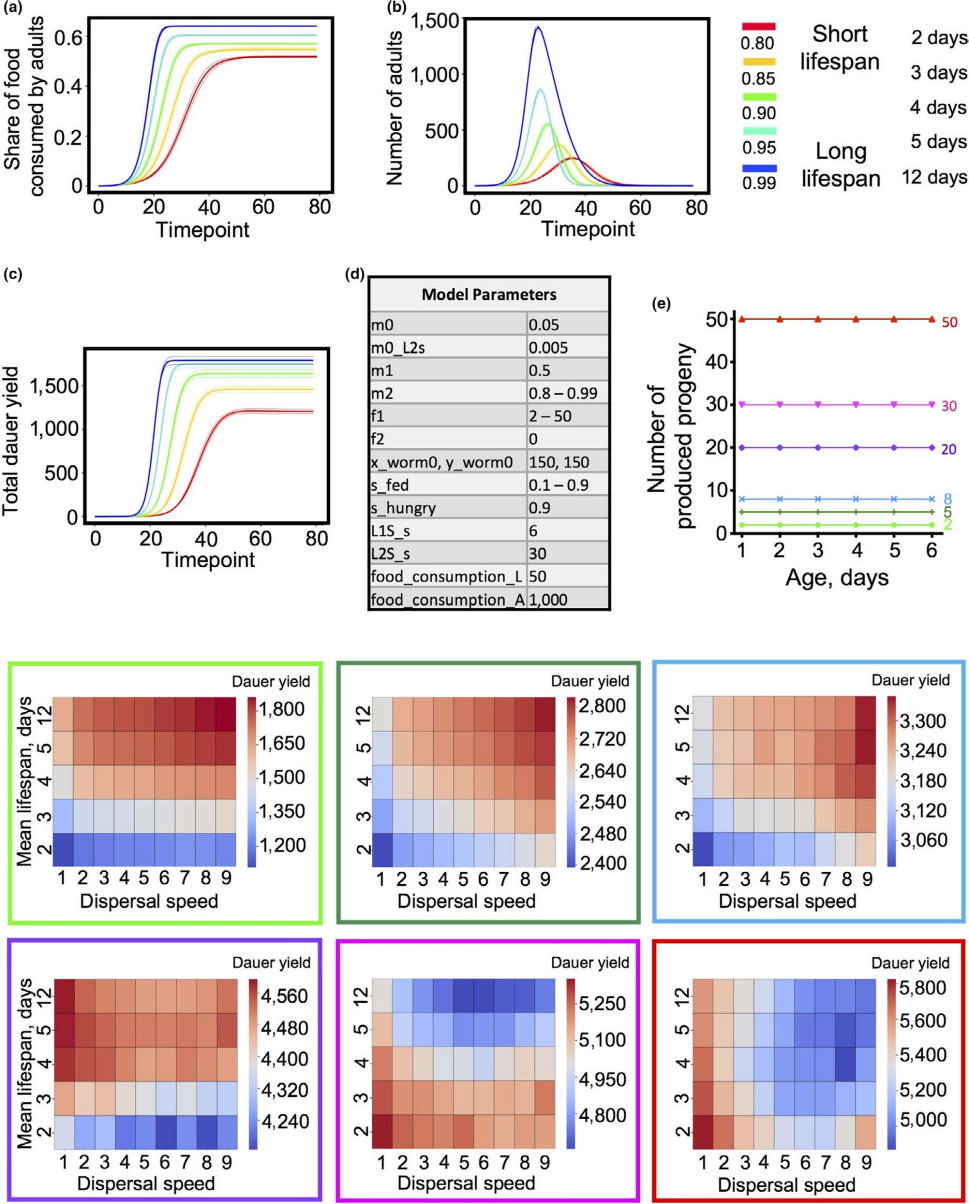
Progeny production rate determines lifespan effect on fitness when there is no reproductive decline. For (a)–(d) and (e) first heatmap (in light green), comparison of 45 dispersal speed × lifespan combinations are shown for the condition when progeny production rate equals 2 and there is no reproductive decline. The color code for all combinations is shown on the side panel. (a) The proportion of food consumed by adults by a timepoint. (b) The numbers of live adults at a timepoint. (c) The sums of dauers produced by a timepoint. (d) Model parameters. (e) Heat maps representing the sums of dauers produced by the end of the simulations for 45 lifespan × dispersal speed combinations for different progeny production rates. The initial scheme (top; right) illustrates reproductive schedules for different progeny production rates when there is no reproductive decline

As an initial negative control, we measured the relative fitness of the 45 genotypes in the absence of any decline in reproduction with age, expecting to see no adaptive death. The number of progeny produced per day was varied from two up to 50; *C. elegans* fecundity in the wild is unknown, but is likely to be lower on average than that measured under nutritionally replete conditions in the laboratory (~300 progeny per worm). Given two progeny/day, the share of the available food consumed by adults was higher in longer‐lived genotypes (Figure 3a), and dauer number peaked shortly after that of adults (Figure 3b,c; Videos S1 and S2). Increasing progeny production rate led to increased colony fitness (Figure S2a), which could be at least partly attributable to an increase in the proportion of food consumed by larvae (Figure S2b). The reduction in relative food consumption by fecund adults is expected given the higher ratio of larvae to adults.

Consistent with the Travis and Markov models, in the absence of reproductive decline, but at low progeny production rates, the genotype with the slowest aging rate was the fittest, leading to the largest yield of both adults and dauers (Figure 3b,c). However, against expectation, from eight progeny/day upwards, shorter life increasingly enhanced inclusive fitness (Figure 3e). One possibility is that this reflects reduction in food consumption by older adults which would otherwise lead to larval food deprivation. Consistent with this, reduction of adult lifespan reduces the proportion of food consumed by adults (Figure S3) and increases the proportion of food that is consumed by larvae, for example from 36% to 48% at two progeny/day and from 63% to 73% at 50 progeny/day. This suggests that earlier adult death increases the proportion of food that is converted into dauer biomass.

Why might shorter life promote fitness in the absence of programmed reproductive decline? Perhaps because of the reproductive decline that will occur anyway in a high proportion of adults due to food depletion, which will cause starvation of newly produced L1s. We postulate that progeny production rates of >8 lead to an increasing proportion of adults where food consumption is futile, that is unproductive in terms of dauer yield. Programmed death in this group could promote fitness through consumer sacrifice, by increasing food availability for animals where food consumption is productive (e.g., existing L1s).

As fecundity increased above 8 and earlier death began to increase fitness, the effect of population viscosity on colony fitness (dauer yield) also changed, from reducing fitness to increasing it (Figure 3e; see shift of red from right to left when progeny increases from 8 to 20). This could reflect increased adult food consumption at high population viscosity due to fewer adults leaving the food patch, which deprives larvae of food and creates a fitness benefit to earlier adult death. Consistent with this interpretation, high population viscosity slightly increases relative adult food consumption at low reproductive rates (2–5; Figure S3a,b). At reproductive rates of 30–50 progeny/day, the optimal dispersal speed was 1, and higher viscosity led to reduced relative adult food consumption (Figure S3c,d). This could reflect adults competing for food with large numbers of larvae at the same location.

### 2.3 Early death increases fitness when reproduction is curtailed

**Figure 4:**
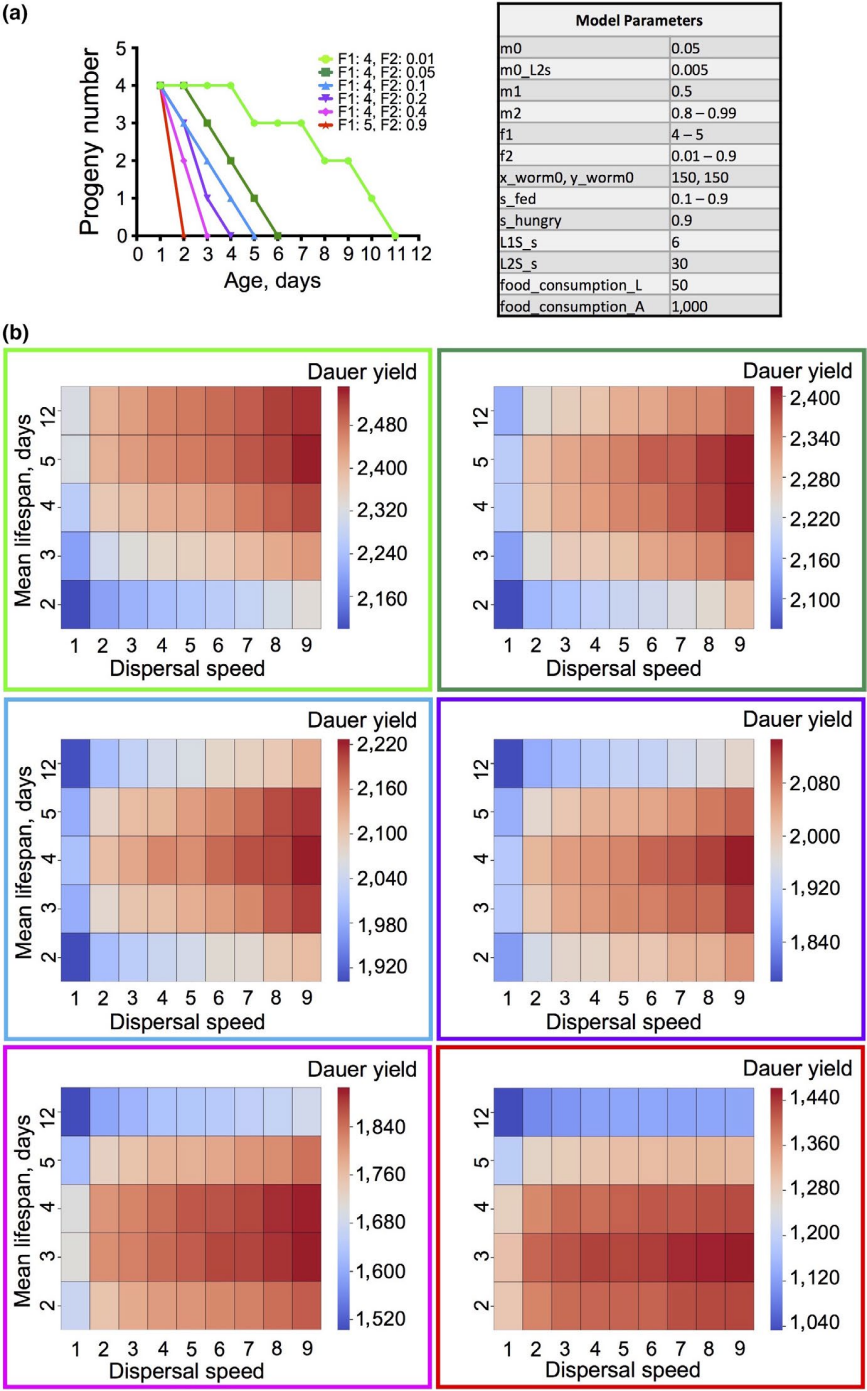
Programmed death can increase fitness given short reproductive span. (a) The scheme shows how F1 and F2 parameters model different curtailed reproductive schedules. The table represents all the parameters for the simulations. (b) Heat maps representing the sums of dauers produced by the end of the simulations for 45 lifespan × dispersal speed combinations for the different progeny production schedules shown in (a)

The condition of protandrous hermaphroditism in *C. elegans* leads to early cessation of reproduction, which could facilitate the evolution of adaptive death (Lohr et al., 2019). To model the effects of the evolution of protandry from a gonochoristic ancestor, we tuned down the reproductive schedule from 30 progeny per adult over 10 days, down to 4 progeny on day 1 only (Figure 4a). Results showed that at the longest reproductive span, the fittest genotypes are the longest lived and least viscous (Figure 4b). However, as reproductive span falls, a shorter lifespan (3–4 days) becomes optimal for fitness. The same set of 100 simulations were performed for the shortest reproductive span (4 progeny on day 1 only) as part of a later test and gave very similar results (Figure 6b, magenta), verifying this finding. Thus, shorter lifespan can increase colony fitness more where reproductive span is shorter (given moderate levels of fecundity).

Notably, where reproduction declined with age, adaptive death was favored by *lower* viscosity at all reproductive spans (Figure 4b). Comparing results in Figures 3e and 4b could imply that when reproductive rate is low, it is always beneficial to reduce food consumption by postreproductive adults, either by their departure from the food patch or by their death. Consistent with this interpretation, for all reproductive spans the proportion of the food consumed by adults drops, though only slightly (typically 0.5%–1.5%), as dispersal rate increases (Figure S4a–g).

One possibility is that the observed colony fitness benefits of shorter life are peculiar to the particular conditions of the model. To probe this, four of the normally invariant model parameters were varied: the number of founder worms, the size of the grid, amount of food in the food patch and the morphology of the food patch. To detect both increases and decreases in optimal lifespan, a control parameter set was selected in which an intermediate lifespan was optimal (including 4 progeny on day 1 only). Optimal lifespan was not altered by increasing the number of founders from 1 to 3, 5 or 7 (Figure S7), or changing of the grid size from 300 × 300 to 200 × 200 or 500 × 500 cells (Figure S8), and was only slightly altered by a flatter or steeper food patch morphology (Figure S9). By contrast, optimal lifespan was markedly reduced by a fivefold increase in food patch size and modestly increased by halving the food patch size (Figure S10), suggesting that *C. elegans* lifespan is optimized to a particular food patch size. These tests suggest that the adaptive value of death is not particularly sensitive to parameters that are constant in the other simulations.

### 2.4 Increasing early reproduction favors adaptive death

**Figure 5:**
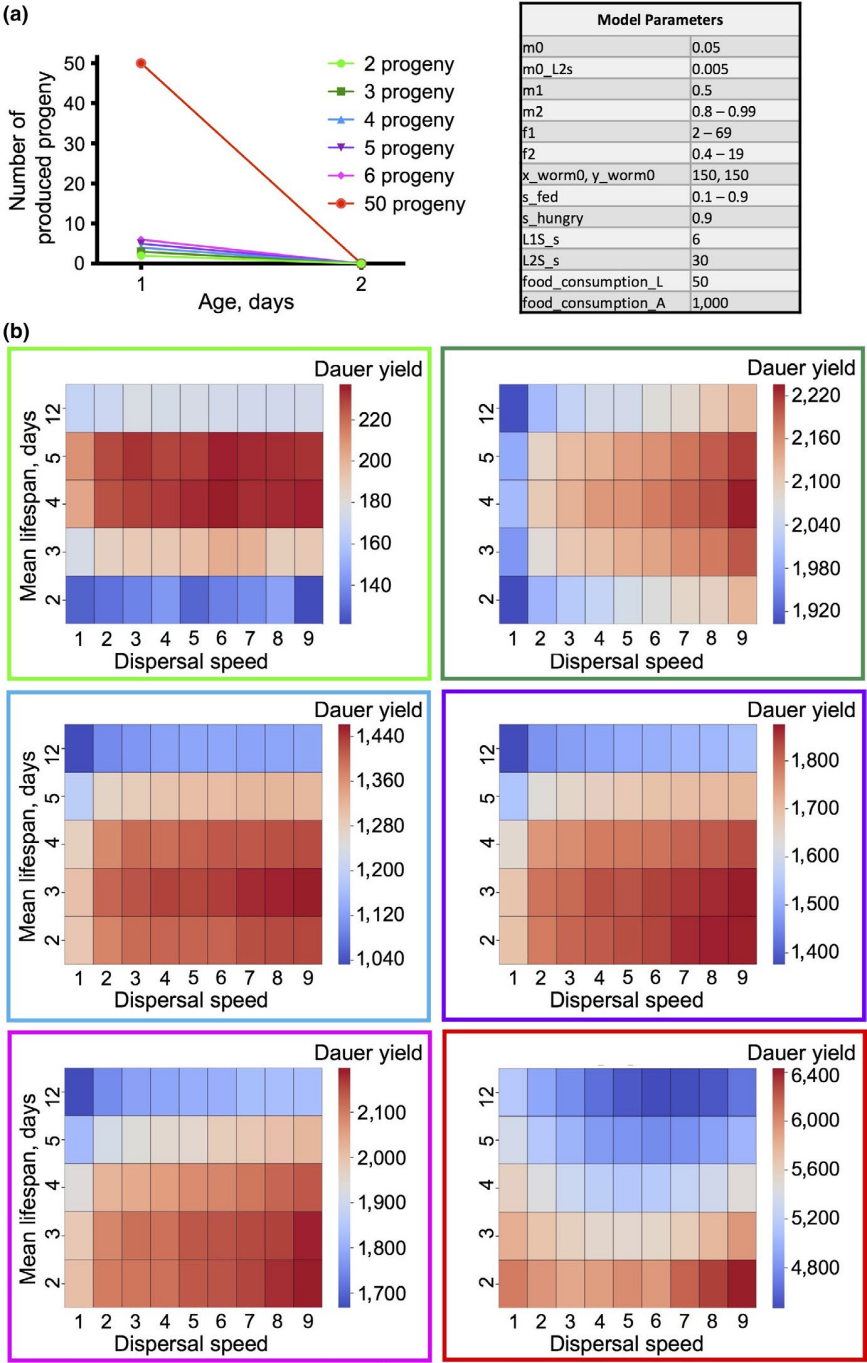
Increasing early reproduction results in a shorter optimal lifespan. (a) The scheme shows reproductive schedules with different reproductive rate when reproduction happens only on day 1. The table represents all the parameters for the simulations. (b) Heat maps representing the sums of dauers produced by the end of the simulations for 45 lifespan × dispersal speed combinations for the different progeny production schedules shown in (a)

That early death increases fitness when reproductive span is short is consistent with the occurrence of consumer sacrifice. According to this interpretation, postreproductive parents compete with their progeny for a limited food supply, such that programmed death in older adults increases food availability for progeny, thereby increasing dauer yield (Lohr et al., 2019). To test this further, we limited reproduction to day 1 of adulthood and increased competition between parents and larvae by increasing reproductive output from 2 to up to 50 progeny per worm. Results of simulations showed that increasing larva to adult ratio does indeed cause shorter adult lifespans to be optimal for fitness (Figure 5). Optimal mortality rate increases from M2 = 0.9–0.95 given 2 larvae to 0.85–0.8 at 5 progeny and above. This is consistent with action of consumer sacrifice.

Interestingly and unexpectedly, an output of 50 progeny only on day 1 gave a greater colony yield (6,400 dauers) than 50 progeny per day with no age decline in reproduction (5,800 dauers; compare Figures 3e, and 5b, bottom right panel). To verify this surprising finding, a second set of simulations were carried out, with similar results (day 1 reproduction only: 6,300 dauers; no reproductive decline: 5,900 dauers). This suggests that an early burst of progeny production is optimal for colony fitness. A possible reason for this is that sustained fecundity leads to too much food competition. Such competition could be between larvae or between adults and larvae, but the former is more likely, since sustained reproduction only slightly reduced the proportion of food consumed by adults (27% compared to 30% with reproduction on day 1 only; Figure S5a; highest viscosity in each case). Notably, the proportion of animals reaching the dauer stage was 8.5% given reproduction on day 1 only, but only ~3% in the absence of reproductive decline (Figure S5b). This implies that sustained reproduction increases the proportion of larvae that starve before reaching the dauer stage. Thus, the behavior of our in silico worm supports a new view of how *C. elegans* life history combines a brief burst of reproduction with short lifespan to optimize fitness in the context of existence as clonal colonies on small food patches.

### 2.5 Where adults are greedier, it is better for them to die sooner

**Figure 6:**
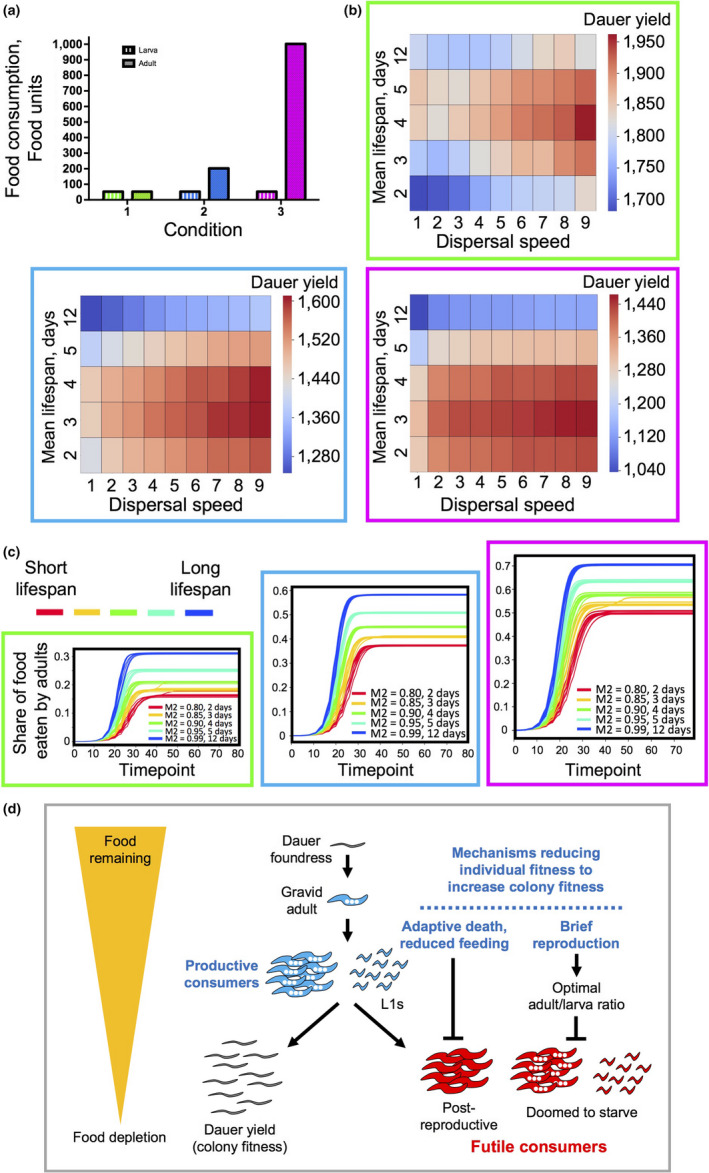
Shorter life promotes fitness more when adults are greedier. Increasing food consumption by adults favors adaptive death. (a) Scheme showing food consumption rate combinations for adults and larvae. In these simulations, adults produced 4 progeny only on day 1. (b) Heat maps depicting final dauer yield for the 3 levels of adult food consumption shown in (a). (c) Share of food consumed by adults at different food consumption rates. Color code for 3 adult food consumption rates (surrounding box) as in (a). Color code for 5 lifespans shown in (c). (d) Summary of conclusions based on behavior of virtual *Caenorhabditis elegans*. Colony fitness is optimized by maximizing efficiency of production of dauers from available food. Futile food consumption, that is that not leading to dauer production, is minimized by reducing individual fitness in order to increase colony fitness. This involves programmed death of adults (consumer sacrifice‐type adaptive death) and optimization of population structure to avoid an excess of early larvae that are doomed to starve by means of a brief, early burst of reproduction

As a further test of the possible action of a consumer sacrifice mechanism, we varied relative adult and larval food consumption rates. Our expectation was that the more food (relatively) that adults consumed, the greater would be the fitness benefits of their early death. In these simulations, we specified a short reproductive span (4 progeny on day 1 only) and a dauer lifespan of 30 days. As in all previous simulations, larval food consumption (food units/day) was 50, but adult food consumption was varied from 50 up to 1,000. As predicted, increasing food consumption increased fitness at shorter lifespans and reduced optimal lifespan (Figure 6a,b). Moreover (and as expected), the share of the food consumed by adults increased when adult food consumption rate was higher, rising from 0.15–0.3 to 0.5–0.7 (Figure 6b,c). Thus, in our virtual worm, adult greed favors the evolution of shorter lifespan.

To confirm this, we varied adult to larval food consumption ratio in a different way: by tuning larval food consumption rate from 25 up to 500 while keeping adult food consumption constant at 1,000 (Figure S6a,b). As predicted, the adaptive value of short life declined with increasing larval food consumption (i.e., with reduced relative adult food consumption; Figure S6b). The share of adult food consumption decreased from 0.8 to 0.2 (blue lines) for the longest lifespan and from 0.6 to 0.05 (red lines) for the shortest lifespan as relative larval food consumption is increased from 1/40 (25 food units) to ½ (500 food units; Figure S6c,d).

### 2.6 Age‐related decline in adult food consumption can promote colony fitness

In aging *C. elegans*, the rate of feeding (pharyngeal pumping) declines rapidly from day 4 of adulthood onwards (Huang, Xiong, & Kornfeld, 2004). Our findings raise the possibility that natural selection has favored an age decline in food consumption in *C. elegans* because it reduces futile food consumption, thereby increasing colony fitness. To test this possibility, we incorporated into our model an age decline in food consumption approximating that described by Huang et al., but this did not increase dauer yield (Figure S11). However, under wild conditions pumping rate, like mortality rate, may change more rapidly with age. To probe this, we introduced an earlier, precipitous decline in feeding rate, from 100% on day 2 to 0 on day 3. This increased dauer yield, consistent with adaptive aphagy, and also suppressed the benefit of early death, consistent with consumer sacrifice (Figure S11). Thus, it is possible that the age decline in feeding rate in *C. elegans* is to some extent a maturational rather than a senescent change. For a summary figure comparing relative colony fitness (dauer yield) across multiple conditions, see Figure S12.

## 3 DISCUSSION

A full understanding of *C. elegans* biology should include an account of how it optimizes fitness, but how should fitness be understood? We have proposed that *C. elegans* life history has evolved to maximize colony fitness, measurable in terms of number of dauer colony propagules. But what is the best way for the individual *C. elegans* life history to maximize dauer yield? One strategy would be to live as long as possible in order to lay as many eggs as possible. However, the behavior of our virtual worm presents a different picture, in which key to maximizing fitness is minimizing food consumption that does not promote dauer production.

Our findings suggest the importance of three types of futile food consumption: first, as initially proposed, food consumption by postreproductive adults; second, food consumption by reproductive adults in colonies approaching food depletion, where progeny cannot reach the dauer stage; and third, food consumption by early larvae who are doomed due to impending food depletion (summary scheme, Figure 6d). Each form of futile consumption can be minimized by life history optimization. Futile adult food consumption can be avoided by adaptive death not only in postreproductive adults, as previously proposed (Lohr et al., 2019), but also in adults with continuous reproduction (Figures 3e, S2 and S3) since on a dwindling food patch they become postreproductive as far as dauer yield is concerned (Figure 6d). Futile food consumption in postreproductive adults can also be reduced by a programmed age decline in food consumption rate (Huang et al., 2004), which our findings suggest could have evolved to promote colony fitness (Figure S11).

Futile larval food consumption can also be reduced by other means. It was previously suggested that *C. elegans* actively limit their brood size in order to limit population growth and conserve resources (Hughes et al., 2007; Kocsisova, Kornfeld, & Schedl, 2019). Our findings suggest instead that curtailing individual reproduction could paradoxically increase dauer yield. Avoiding futile food consumption in early larvae involves a complex challenge for the lucky dauer larva that encounters a food patch and initiates a clonal population, as follows. It needs to generate a population of adults just large enough that there is sufficient food for as many of their progeny as possible to reach the dauer stage (Figure 6d). Too high fecundity leads to increased futile early larval food consumption; consistent with this, in the virtual worm dauer yield was greater in a genotype with a burst of early reproduction than in one where reproduction was sustained (Figures 3e and 5b). This conclusion will chime well with anyone who has worked with *C. elegans*, where establishing a stock plate on an *Escherichia coli* lawn with three worms leads within a few days to a mixed stage population including second‐generation adults, whereas starting with, say, 10 worms yields a population of mainly starved, early stage larvae. That *C. elegans* might limit its brood size to reduce competition for resources among progeny was suggested previously by Hughes et al. (2007).

That increasing adult food consumption increased the fitness benefits from early death (Figure 6b) provides a good illustration of the action of consumer sacrifice. A negative correlation between optimal lifespan and probability of exhausting resources by adults was also observed in a previous modelling study (Werfel, Ingber, & Bar‐Yam, 2015). Notably, Werfel et al*.* also found that age‐related reproductive decline is not required for shorter life to be adaptive, as observed here (Figure 3e).

The behavior of the model shows how continued adult survival could have antagonistic effects on *C. elegans* colony fitness: promoting it by allowing production of more eggs and reducing it by increasing futile food consumption. When progeny production rate is low (<8), longer lifespan is better because the fitness benefits of laying more eggs outweigh the costs in terms of futile food consumption. By contrast, when progeny production rate is high (>8), long life can increase the proportion of futile food consumption, particularly by early larvae doomed to starve before reaching the dauer stage, such that overall fitness is reduced. Thus, shorter life can increase fitness even in genotypes without a set age decline in reproduction, probably by reducing progeny production (Figures 3e and 6d). This suggests that consumer sacrifice adaptive death could also have evolved in gonochoristic species of *Caenorhabditis* where reproductive span is not limited by protandry.

Our finding that increased fitness from early death can occur when population viscosity is low does not conflict with evolutionary theory. The prediction that adaptive death is beneficial where populations are viscous is based on population genetic reasoning (avoidance of exploitation of altruists by parasitic egoists; Hamilton, 1964; Lohr et al., 2019; Figure 1a). But this aspect of life history evolution is not included in our model. In contrast to earlier studies (Markov, 2012; Travis, 2004; Werfel et al., 2015), viscosity in our model affects efficiency of resource conversion into dauers rather than resource inheritance as defined in those studies (as we have only one genotype in each simulation).

How life‐like is the model described here with respect to *C. elegans*? Clearly, a concern is that properties of the model and of *C. elegans* will differ such that any conclusions drawn here could be inapplicable to real *C. elegans*. Here, a problem is that for key parameters of the model, authentic values for wild *C. elegans* are lacking, including reproductive schedule, fecundity, larval mortality and lifespan. These values are likely to change as colony life history unfolds. One may deduce that in a boom‐and‐bust setting, the largest number of adults will exist at the point of maximum colony size, shortly prior to food depletion. From this and the presence of pathogenic microbes (Schulenburg & Félix, 2017), it may be safely assumed that mean lifespan of adult *C. elegans* in the wild is far shorter than that measured under optimal culture conditions, perhaps only 1–3 days rather than 2–3 weeks (Klass, 1977). This predicts that programmed, adaptive death mechanisms would need to be activated within days of reproductive maturity to produce any fitness benefits. Consistent with this, a number of degenerative changes have been observed in *C. elegans* during the first week of adulthood; reviewed by Lohr et al. (2019). This includes a collapse in protein folding homeostasis starting at the onset of reproduction, only 6 hr after the L4 to adult molt (Labbadia & Morimoto, 2015). Thus, arguably, inclusion in our model of lifespans as low as 2 days and brood sizes as low as 2 is not far‐fetched.

In our simulations, increased reproduction increased the adaptive value of shorter lifespan (Figures 3e and 5b). Negative correlations between reproduction and lifespan are widespread in nature (Partridge, Gems, & Withers, 2005; Stearns, 1992). Such correlations can result from various forms of trade‐off involving ecological and physiological costs (Speakman, 2008). The behavior of virtual *C. elegans* describes a reproduction–lifespan trade‐off where increased reproduction increases food competition between parents and offspring, thereby increasing the fitness benefits of consumer sacrifice‐type adaptive death. Whether consumer sacrifice‐type reproduction–lifespan trade‐offs actually occur in the natural world is a question for future investigation.

In conclusion, the behavior of our computer model suggests that individual *C. elegans* life history can evolve to maximize colony fitness by minimizing futile food consumption. It does this not only by consumer sacrifice‐type adaptive death, as we initially proposed, but also through a reproductive schedule that generates an optimal population structure (Figure 6d). This joins a growing list of strategies by which androdioecious *Caenorhabditis* maximize early reproduction and population growth, including an early switch from sperm to egg production (Hodgkin & Barnes, 1991; Poullet et al., 2016), reduction of male frequency by various mechanisms (Garcia, LeBoeuf, & Koo, 2007; LaMunyon & Ward, 1997; Stewart & Phillips, 2002; Yin & Haag, 2019) and even earlier onset of reproduction in adults born from the first eggs (Perez, Francesconi, Hidalgo‐Carcedo, & Lehner, 2017). Plausibly, reduction of individual fitness to minimize futile food consumption can only increase fitness in organisms such as *C. elegans* (colonial and with a dispersal form). However, it is interesting to consider whether food use efficiency might influence life history evolution in other types of organism, for example nonclonal, lacking a dispersal form or on extended food sources.

We have suggested that adaptive death is a peculiarity of organisms existing as viscous, clonal populations and that mutations extending *C. elegans* lifespan may act by suppressing adaptive death. But mutations in the insulin/IGF‐1 signalling (IIS) pathway, for example, increase lifespan not only in *C. elegans*, but also in higher taxa (e.g., in *Drosophila*, mouse). To explain this, we previously suggested that genes with smaller effects on senescent pathology in other taxa were co‐opted to promote adaptive death in *C. elegans* (Lohr et al., 2019). Consistent with this, the magnitude of effects of IIS on lifespan in other animal groups is far less than in *C. elegans*. For example, mutation of the phosphatidylinositol 3‐kinase catalytic subunit gene increases median lifespan by up to ~10‐fold in *C. elegans* but only ~1.07‐fold and ~1.02‐fold in *Drosophila* and mice, respectively (Ayyadevara et al., 2008; Foukas et al., 2013; Slack, Giannakou, Foley, Goss, & Partridge, 2011).

## 4 EXPERIMENTALPROCEDURES

### 4.1 Further details of the model

When designing the model, in order to reduce computer processing time, *C. elegans* life history features were simplified and approximated. Our aim was not to generate the most realistic model possible, but to explore the feasibility of creating a *C. elegans*‐like model and to test whether in such an approximation fitness benefits of adaptive death could be observed. The model provides a starting point for designing further iterations that more closely resemble *C. elegans* life history.

In more detail, simulations were performed on a 300 × 300 grid containing at its centre food distributed as a 2D Gaussian. During simulations, the food source was loaded as an CSV file, space_central_gauss.csv which can be found at Mendeley Data: Data/Scripts/scripts/data/, which is a 300 × 300 table containing values of food units. Space_central_gauss.csv was created using Create_space.py script.

In our simplified model, each in silico worm was created as a Python object of class “Organism” defined in the file worms2.py. In the worms2.py script, we also defined functions determining how “worms” transition from one developmental stage to the next, how probability of survival for each age is determined, and how they reproduce and move. The simulation process was executed using mainloop_def_leg3_fast.py script, where all the functions defined in worms2.py are applied to each in silico worm at every timepoint. Time was determined in discrete steps and all individuals were tracked.

As described above, for the sake of simplicity, we reduced the number of larval stages from 4 to 2. Each worm appears at the L0 stage (or egg) and at the next timepoint “develops” into an L1 (first stage larva). If food is available, it then develops into an L2 and then into an adult during the next successive timepoints. Adults also become older with each timepoint (each object in the stage “adult” has an attribute “age”). At each timepoint, the script runs through the current population and applies all the functions from the script worms2.py in the following order. First, “worms” consume food. If the number of food units is higher than the consumption rate of an individual located in the cell, then the individual is flagged as fed, and if not as starved. Second, a staging function called “time” (in worms2.py) is applied to each individual. The transformations happen according to the table below (see Figure S13a,b).

**Table:** 

Stage and fed status	New stage	Comment
L0	L1	
L1 and fed	L2	
L1 and starved	L1_arrested_	
L1_arrested_ and starved	L1_arrested_	The number of days in the starved state is counted. If it reaches the limit that is set in L1s_s parameter (it was 6 for all simulations), the individual dies
L2 and fed	Adult	
L2 and starved	Dauer	
Dauer and fed	Adult	
Dauer and starved	Dauer	The number of days in the starved state is counted. If it reaches the limit that is set in L2s_s parameter (it was 30 for all simulations), the individual dies
Adult and fed or starved	Adult	For adults age is counted and it grows by one with every timepoint. Age determines mortality rate, fecundity (and in some simulations food consumption rate)
Dead	Dead	

After stage determination, the function “survive” (defined in worms2.py) is applied to each individual. All L0s always survive. For all other individuals, whether they die or not is determined randomly according to their probability of death. The probability of death for L1, L1_arrested_, L2 is set .05, for dauers −.005. For adults, it depends on age and is determined by a formula: *p*_death_(age) = 1 − M2^(M1 + age)^, where M1 and M2 determine how quickly *p*_death_(age) approaches 1.

Each individual is located at a particular cell on the grid. Next, each individual changes its location according to the function “move” (determined in worms2.py). The distance moved is determined by the fed status of the individual and limited with s_fed and s_hungry parameters when an in silico worm is fed or starved accordingly. Both direction and the distance of movement are determined randomly for *x* and *y* coordinates (Figure S13c). For example, if s_fed = 0.5, the *x* or *y* coordinate can change from −5 to 5 cells away from the initial one; if initial coordinates are (150, 150), the next position can be (146, 155) or (150, 151). “Worms” cannot leave the grid, so if *x* or *y* exceed the limit, they are set to the limit. For example, if *x* is 305, it is set to 300 (when the grid size is 300 × 300).

Next, each individual that reached the adult stage reproduces. The number of progeny is determined by the function “progeny_n” defined in worms2.py. This function calculates the number of progeny based on F1 and F2 parameters according to the formula Number of progeny (age) = F1 − F2*age^2.5^. The function number of progeny (age) was set to equal zero in all cases when it was below zero. Each individual reproduces according to the function “reproduce” (defined in worms2.py), and the determined number of progeny appears at the same location as the parent.

For each set of parameters, we measure colony fitness as the cumulative sum of dauers produced by simulation when the food is exhausted. In colony fitness assays, we initially measured not only the cumulative sum of dauers produced by a particular timepoint, but also peak dauer production. The inclusion of the latter metric was based on knowledge of *C. elegans* ecology: given that dauer dispersal by animal vectors is aided by dauer tower formation (Felix & Duveau, 2012), we reasoned that peak dauer number is a function of tower size which is, in turn, a metric for dispersal capacity and fitness. However, results for sum and peak dauer numbers were very similar (data not shown), and so, only the former are reported here. Another possible colony fitness metric is *rate* of dauer production; this was not included here in order to limit the complexity of the analysis.

In simulations, because mortality was determined probabilistically (stochastically), in some cases, population establishment failed due to the death of the initiating larva. Simulations with this outcome were excluded, and mean fitness calculated using only those outcomes with non‐null fitness. For most trials, an initiating population size of 1 was selected for simplicity's sake; by one estimate based on study of wild *C. elegans,* colonies (subpopulations or demes) are initiated by 3–10 individuals (Richaud et al., 2018).

Besides the closer resemblance of our model to *C. elegans*, significant differences in previous models (Markov, 2012; Travis, 2004) include (a) the presence of competing genotypes on the same grid; (b) each cell on the patch could be occupied by only one individual; and (c) the Travis (2004) model included mutation and selection. Regarding (a): given the hypothesis that adaptive death will be beneficial under viscous conditions (Figure 1a), we opted instead to measure individual colony fitness rather than having different genotypes compete for the same patch. This means that effects of population viscosity in our model should be interpreted in a different way than for previous models: because we have only one genotype per food patch, dispersal speed determines efficiency of conversion of food into dauers rather than proportion of food consumed by kin.

### 4.2 Coding of the model

Python 3.6 was used to implement the model and all scripts are available in Data S1.

### 4.3 Running the simulations

Simulations were run using the UCL Myriad High Performance Computing Facility (Myriad@UCL) and associated support services. 100 repeat simulations for each condition were performed. To parallelize running between various nodes, additional bash scripts were written. Worms2.py contains the object oriented model of *C. elegans*. The run3_fast.py contains parameters which were changed before the run. Other python scripts were necessary to perform analyses, copy data and create graphs. Bash scripts were used to run parallel simulations in the Myriad cluster. After parameters were updated in run3_fast.py, the master script 8.sh was set to run 100 repeats for each of the 45 different conditions.

## CONFLICT OF INTEREST

The authors declare no competing interests.

## AUTHOR CONTRIBUTIONS

DG and ERG conceived of the project. ERG constructed the in silico worm and performed the simulations. DG and ERG wrote the manuscript.

## Supporting information

Supplementary Material

Video S1

Video S2

## Data Availability

Raw as well as analysed data, scripts and graphs were deposited in Mendeley Data (Galimov & Gems, 2020).

## References

[acel13141-bib-0001] Arantes‐Oliveira, N. , Berman, J. R. , & Kenyon, C. (2003). Healthy animals with extreme longevity. Science, 302, 611. 14576426 10.1126/science.1089169

[acel13141-bib-0002] Ayyadevara, S. , Alla, R. , Thaden, J. J. , & Shmookler Reis, R. J. (2008). Remarkable longevity and stress resistance of nematode PI3K‐null mutants. Aging Cell, 7, 13–22. 17996009 10.1111/j.1474-9726.2007.00348.x

[acel13141-bib-0003] Banfield, K. , Gomez, T. , Lee, W. , Clarke, S. , & Larsen, P. (2008). Protein‐repair and hormone‐signaling pathways specify dauer and adult longevity and dauer development in *Caenorhabditis elegans* . Journals of Gerontology. Series A, Biological Sciences and Medical Sciences, 63, 798–808. 10.1093/gerona/63.8.798 PMC2630856 18772467

[acel13141-bib-0004] Barriere, A. , & Felix, M.‐A. (2007). Temporal dynamics and linkage disequilibrium in natural *Caenorhabditis elegans* populations. Genetics, 176, 999–1011. 17409084 10.1534/genetics.106.067223 PMC1894625

[acel13141-bib-0005] Baugh, L. (2013). To grow or not to grow: Nutritional control of development during *Caenorhabditis elegans* L1 arrest. Genetics, 194, 539–555. 23824969 10.1534/genetics.113.150847 PMC3697962

[acel13141-bib-0006] Cassada, R. C. , & Russell, R. L. (1975). The dauerlarva, a post‐embryonic developmental variant of the nematode *Caenorhabditis elegans* . Developmental Biology, 46, 326–342. 1183723 10.1016/0012-1606(75)90109-8

[acel13141-bib-0007] Cutter, A. D. (2015). *Caenorhabditis* evolution in the wild. BioEssays, 37, 983–995. 26126900 10.1002/bies.201500053

[acel13141-bib-0008] Dytham, C. , & Travis, J. M. J. (2006). Evolving dispersal and age at death. Oikos, 113, 530–538.

[acel13141-bib-0009] Ellis, R. , & Lin, S.‐Y. (2014). The evolutionary origins and consequences of self‐fertility in nematodes. F1000Prime Reports, 6, 62. 25165561 10.12703/P6-62 PMC4126538

[acel13141-bib-0010] Felix, M.‐A. , & Duveau, F. (2012). Population dynamics and habitat sharing of natural populations of *Caenorhabditis elegans* and *C. briggsae* . BMC Biology, 10, 59. 22731941 10.1186/1741-7007-10-59 PMC3414772

[acel13141-bib-0011] Foukas, L. , Bilanges, B. , Bettedi, L. , Pearce, W. , Ali, K. , Sancho, S. , … Vanhaesebroeck, B. (2013). Long‐term p110alpha PI3K inactivation exerts a beneficial effect on metabolism. EMBO Molecular Medicine, 5, 563–571. 23483710 10.1002/emmm.201201953 PMC3628103

[acel13141-bib-0012] Frezal, L. , & Felix, M.‐A. (2015). *C. elegans* outside the Petri dish. Elife, 4, e05849. 10.7554/eLife.05849 PMC4373675 25822066

[acel13141-bib-0013] Galimov E. R. , Gems D. (2020). Shorter life and reduced fecundity can increase colony fitness in virtual C. elegans. Mendeley Data, V1 10.17632/9h9dyyxc49.1 PMC7253062 32301222

[acel13141-bib-0014] Galimov, E. R. , Lohr, J. N. , & Gems, D. (2019). When and how can death be an adaptation? Biochemistry (Moscow), 84, 1433–1437. 31870246 10.1134/S0006297919120010

[acel13141-bib-0015] Garcia, L. R. , LeBoeuf, B. , & Koo, P. (2007). Diversity in mating behavior of hermaphroditic and male‐female *Caenorhabditis* nematodes. Genetics, 175, 1761–1771. 17277358 10.1534/genetics.106.068304 PMC1855125

[acel13141-bib-0016] Gems, D. , & Partridge, L. (2013). Genetics of longevity in model organisms: Debates and paradigm shifts. Annual Review of Physiology, 75, 621–644. 10.1146/annurev-physiol-030212-183712 23190075

[acel13141-bib-0017] Gray, J. M. , Karow, D. S. , Lu, H. , Chang, A. J. , Chang, J. S. , Ellis, R. E. , … Bargmann, C. I. (2004). Oxygen sensation and social feeding mediated by a *C. elegans* guanylate cyclase homologue. Nature, 430, 317–322. 15220933 10.1038/nature02714

[acel13141-bib-0018] Hamilton, W. (1964). The genetical evolution of social behaviour. Journal of Theoretical Biology, 7, 1–52. 5875341 10.1016/0022-5193(64)90038-4

[acel13141-bib-0019] Hodgkin, J. , & Barnes, T. M. (1991). More is not better: Brood size and population growth in a self‐fertilizing nematode. Proceedings of the Royal Society of London, Series B: Biological Sciences, 246, 19–24. 1684664 10.1098/rspb.1991.0119

[acel13141-bib-0020] Hodgkin, J. , & Doniach, T. (1997). Natural variation and copulatory plug formation in *Caenorhabditis elegans* . Genetics, 146, 149–164. 9136008 10.1093/genetics/146.1.149 PMC1207933

[acel13141-bib-0021] Houthoofd, K. , Braeckman, B. , & Vanfleteren, J. (2004). The hunt for the record life span in *Caenorhabditis elegans* . Journals of Gerontology. Series A, Biological Sciences and Medical Sciences, 59, B408–B410. 10.1093/gerona/59.5.b408 15123748

[acel13141-bib-0022] Hu, P. J. (2007). Dauer In The C. elegans Research Community (Ed.), WormBook. http://www.wormbook.org

[acel13141-bib-0023] Huang, C. , Xiong, C. , & Kornfeld, K. (2004). Measurements of age‐related changes of physiological processes that predict lifespan of *Caenorhabditis elegans* . Proceedings of the National Academy of Sciences USA, 101, 8084–8089. 10.1073/pnas.0400848101 PMC419561 15141086

[acel13141-bib-0024] Hughes, S. E. , Evason, K. , Xiong, C. , & Kornfeld, K. (2007). Genetic and pharmacological factors that influence reproductive aging in nematodes. PLoS Genetics, 3(2), e25. 17305431 10.1371/journal.pgen.0030025 PMC1797816

[acel13141-bib-0025] Johnson, T. E. , Mitchell, D. H. , Kline, S. , Kemal, R. , & Foy, J. (1984). Arresting development arrests aging in the nematode *Caenorhabditis elegans* . Mechanisms of Ageing and Development, 28, 23–40. 6542614 10.1016/0047-6374(84)90150-7

[acel13141-bib-0026] Kenyon, C. (2010). The genetics of ageing. Nature, 464, 504–512. 20336132 10.1038/nature08980

[acel13141-bib-0027] Kiontke, K. , Félix, M.‐A. , Ailion, M. , Rockman, M. , Braendle, C. , Pénigault, J.‐B. , & Fitch, D. (2011). A phylogeny and molecular barcodes for *Caenorhabditis*, with numerous new species from rotting fruits. BMC Evolutionary Biology, 11, 339. 22103856 10.1186/1471-2148-11-339 PMC3277298

[acel13141-bib-0028] Klass, M. R. (1977). Aging in the nematode *Caenorhabditis elegans*: Major biological and environmental factors influencing life span. Mechanisms of Ageing and Development, 6, 413–429. 926867 10.1016/0047-6374(77)90043-4

[acel13141-bib-0029] Klass, M. R. , & Hirsh, D. I. (1976). Nonaging developmental variant of *C. elegans* . Nature, 260, 523–525. 1264206 10.1038/260523a0

[acel13141-bib-0030] Kocsisova, Z. , Kornfeld, K. , & Schedl, T. (2019). Rapid population‐wide declines in stem cell number and activity during reproductive aging in *C. elegans* . Development, 146, dev173195. 30936182 10.1242/dev.173195 PMC6503983

[acel13141-bib-0031] Kramer, J. , & Meunier, J. (2016). Kin and multilevel selection in social evolution: A never‐ending controversy? F1000Research, 5, 776. 10.12688/f1000research.8018.1 PMC4850877 27158472

[acel13141-bib-0032] Labbadia, J. , & Morimoto, R. (2015). Repression of the heat shock response is a programmed event at the onset of reproduction. Molecular Cell, 59, 639–650. 26212459 10.1016/j.molcel.2015.06.027 PMC4546525

[acel13141-bib-0033] LaMunyon, C. W. , & Ward, S. (1997). Increased competitiveness of nematode sperm bearing the male X chromosome. Proceedings of the National Academy of Sciences USA, 94, 185–189. 10.1073/pnas.94.1.185 PMC19277 8990183

[acel13141-bib-0034] Lohr, J. , Galimov, E. , & Gems, D. (2019). Does senescence promote fitness in *Caenorhabditis elegans* by causing death? Ageing Research Reviews, 50, 58–71. 30639341 10.1016/j.arr.2019.01.008 PMC6520499

[acel13141-bib-0035] Markov, A. (2012). Can kin selection facilitate the evolution of the genetic program of senescence? Biochemistry (Moscow), 77, 733–741. 22817537 10.1134/S0006297912070061

[acel13141-bib-0036] Maynard Smith, J. (1976). Group selection. Quarterly Review of Biology, 51, 277–283.

[acel13141-bib-0037] Niccoli, T. , & Partridge, L. (2012). Ageing as a risk factor for disease. Current Biology, 22, R741–R752. 22975005 10.1016/j.cub.2012.07.024

[acel13141-bib-0038] Partridge, L. , Gems, D. , & Withers, D. J. (2005). Sex and death: What is the connection? Cell, 120, 461–472. 15734679 10.1016/j.cell.2005.01.026

[acel13141-bib-0039] Perez, M. , Francesconi, M. , Hidalgo‐Carcedo, C. , & Lehner, B. (2017). Maternal age generates phenotypic variation in *Caenorhabditis elegans* . Nature, 552, 106–109. 29186117 10.1038/nature25012 PMC5736127

[acel13141-bib-0040] Petersen, C. , Saebelfeld, M. , Barbosa, C. , Pees, B. , Hermann, R. , Schalkowski, R. , … Schulenburg, H. (2015). Ten years of life in compost: Temporal and spatial variation of North German *Caenorhabditis elegans* populations. Ecology and Evolution, 5, 3250–3263. 26380661 10.1002/ece3.1605 PMC4569023

[acel13141-bib-0041] Poullet, N. , Vielle, A. , Gimond, C. , Carvalho, S. , Teotónio, H. , & Braendle, C. (2016). Complex heterochrony underlies the evolution of *Caenorhabditis elegans* hermaphrodite sex allocation. Evolution, 70, 2357–2369. 27501095 10.1111/evo.13032

[acel13141-bib-0042] Richaud, A. , Zhang, G. , Lee, D. , Lee, J. , & Félix, M. (2018). The local coexistence pattern of selfing genotypes in *Caenorhabditis elegans* natural metapopulations. Genetics, 208, 807–821. 29242287 10.1534/genetics.117.300564 PMC5788539

[acel13141-bib-0043] Rose, M. (1991). Evolutionary biology of aging. Oxford, UK: Oxford University Press.

[acel13141-bib-0044] Sawin, E. , Ranganathan, R. , & Horvitz, H. (2000). *C. elegans* locomotory rate is modulated by the environment through a dopaminergic pathway and by experience through a serotonergic pathway. Neuron, 26, 619–631. 10896158 10.1016/s0896-6273(00)81199-x

[acel13141-bib-0045] Schulenburg, H. , & Félix, M. (2017). The natural biotic environment of *Caenorhabditis elegans* . Genetics, 206, 55–86. 28476862 10.1534/genetics.116.195511 PMC5419493

[acel13141-bib-0046] Slack, C. , Giannakou, M. E. , Foley, A. , Goss, M. , & Partridge, L. (2011). dFOXO‐independent effects of reduced insulin‐like signaling in *Drosophila* . Aging Cell, 10(5), 735–748. 21443682 10.1111/j.1474-9726.2011.00707.x PMC3193374

[acel13141-bib-0047] Speakman, J. R. (2008). The physiological costs of reproduction in small mammals. Philosophical Transactions of the Royal Society of London. Series B, Biological Sciences, 363, 375–398. 17686735 10.1098/rstb.2007.2145 PMC2606756

[acel13141-bib-0048] Stearns, S. C. (1992). The evolution of life histories. Oxford, UK: Oxford University Press.

[acel13141-bib-0049] Stewart, A. , & Phillips, P. (2002). Selection and maintenance of androdioecy in *Caenorhabditis elegans* . Genetics, 160, 975–982. 11901115 10.1093/genetics/160.3.975 PMC1462032

[acel13141-bib-0050] Travis, J. M. J. (2004). The evolution of programmed death in a spatially structured population. Journals of Gerontology. Series A, Biological Sciences and Medical Sciences, 59A, 301–305. 10.1093/gerona/59.4.b301 15071072

[acel13141-bib-0051] Werfel, J. , Ingber, D. , & Bar‐Yam, Y. (2015). Programed death is favored by natural selection in spatial systems. Physical Review Letters, 114, 238103. 26196833 10.1103/PhysRevLett.114.238103

[acel13141-bib-0052] Werfel, J. , Ingber, D. , & Bar‐Yam, Y. (2017). Theory and associated phenomenology for intrinsic mortality arising from natural selection. PLoS ONE, 12, e0173677. 28355288 10.1371/journal.pone.0173677 PMC5371302

[acel13141-bib-0053] Williams, G. C. (1966). Adaptation and natural selection. Princeton, NJ: Princeton University Press.

[acel13141-bib-0054] Wilson, D. S. , & Wilson, E. O. (2008). Evolution “for the Good of the Group”. American Scientist, 96, 380–389.

[acel13141-bib-0055] Wood, W. B. (1988). The nematode *Caenorhabditis elegans*. Plainview, NY: Cold Spring Harbor Press.

[acel13141-bib-0056] Yin, D. , & Haag, E. (2019). Evolution of sex ratio through gene loss. Proceedings of the National Academy of Sciences USA, 116, 12919–12924. 10.1073/pnas.1903925116 PMC6601293 31189601

