# Supplementary material for: Shorter life and reduced fecundity can increase colony fitness in virtual Caenorhabditis elegans
Source: Aging Cell. 2020 Apr 16;19(5):e13141. doi: 10.1111/acel.13141 (PMC7253062; doi:10.1111/acel.13141)
Supplement: Supplementary file 1 — Supplementary Material [file ACEL-19-e13141-s001.docx]

**Supplementary Information**

Shorter life can increase colony fitness in virtual *C. elegans*

Evgeniy R. Galimov and David Gems*

Institute of Healthy Ageing, and Research Department of Genetics, Evolution and Environment, University College London, London WC1E 6BT.

*e-mail: david.gems@ucl.ac.uk

**Supplementary Figures**

Figure S1 The relationship between the parameters in the model.

Figure S2 Higher progeny production rate increases larval food consumption share and fitness.

Figure S3 Adult food consumption share when there is no reproductive decline with age.

Figure S4 Adult food consumption share for different age-dependent reproductive spans.

Figure S5 Reproduction on day 1 only increases fitness more than sustained reproduction when reproduction rate is high.

Figure S6 Higher relative larval food consumption reduces the adaptive value of short lifespan.

Figure S7 Fitness benefit of early death is not sensitive to founder worm number.

Figure S8 Fitness benefit of early death is not sensitive to grid size.

Figure S9 Little effect of food patch morphology on optimal lifespan.

Figure S10 Higher amount of food promotes adaptive death.

Figure S11 The decline in food consumption by adults diminishes the value of adaptive death.

Figure S12 Comparison of dauer yield given a variety of conditions.

Figure S13 A more detailed explanation of the model.

**Supplementary Videos**

Video S1 Growth of the dauer population during first 40 time points given no reproductive decline.

Video S2 Depletion of the food source first 40 time points given no reproductive decline.

**Supplementary Data**

Supplementary Data S1 Raw as well as analysed data, scripts and graphs were deposited in Mendeley Data (Galimov, E., & Gems, D. 2019, Shorter life can increase colony fitness in virtual *C. elegans*, Mendeley Data, V1, doi: 10.17632/9h9dyyxc49.1)

**
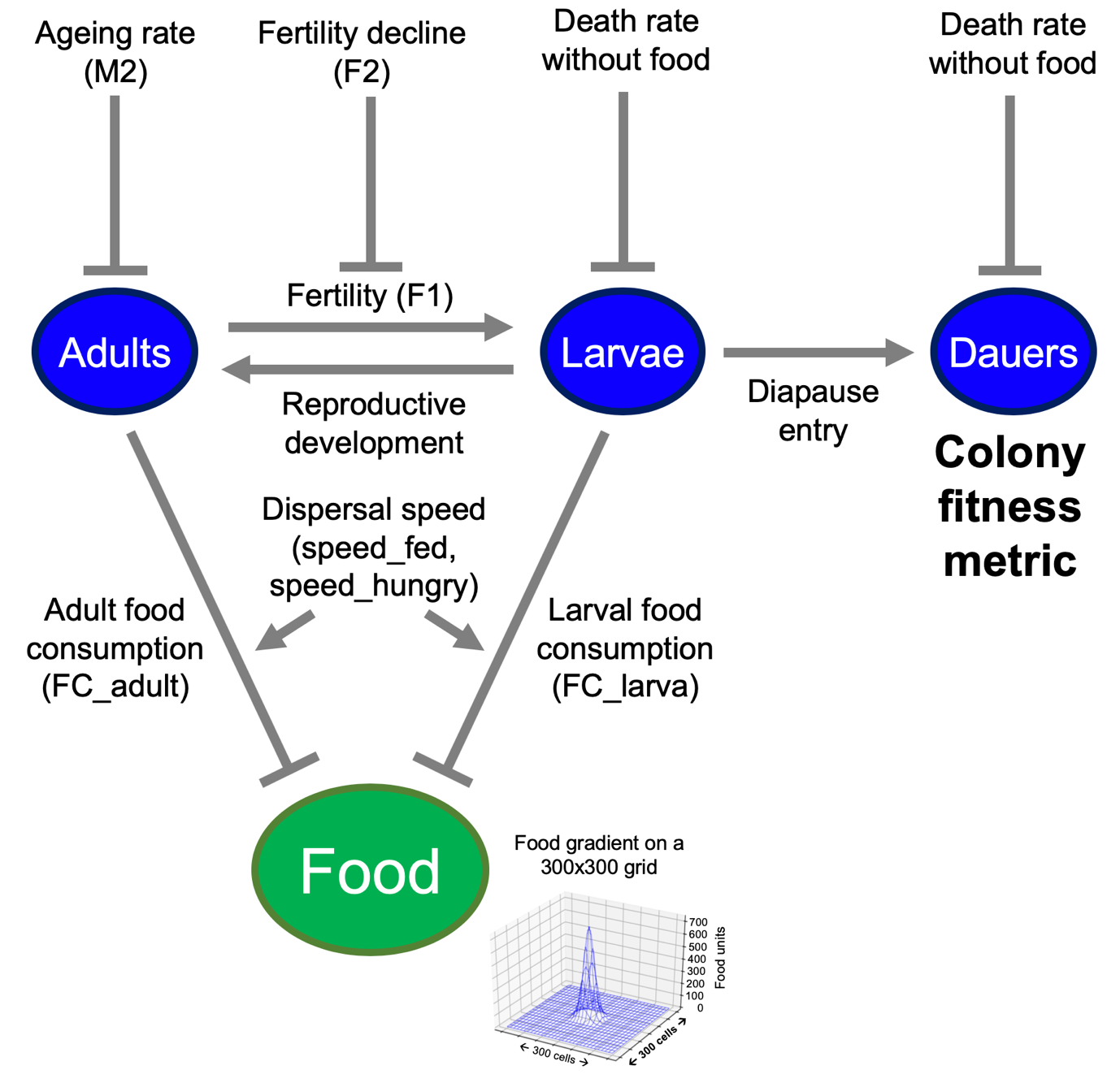
**

**Figure S1** **The relationship between the parameters in the model.** Optimal ageing rate reflects a balance (trade off) between living longer and having more offspring, and living shorter to reduce adult food consumption which may reduce dauer yield.

**
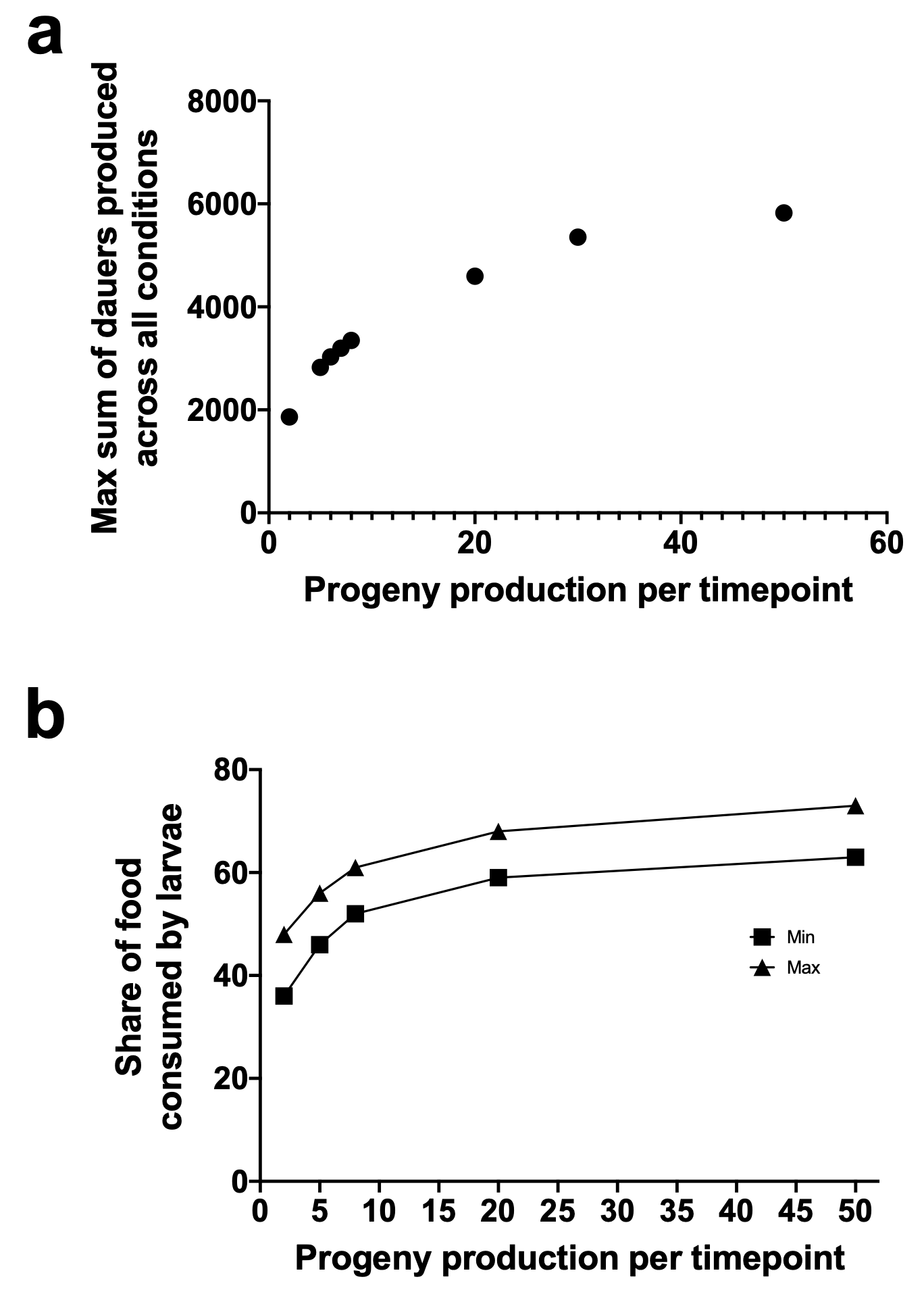
**

**Figure S2 Higher progeny production rate increases larval food consumption share and fitness. a,** Increasing progeny production rate favours fitness when there is no reproductive decline. **b,** High progeny production rate increases the share (proportion) of food consumed by larvae.

**
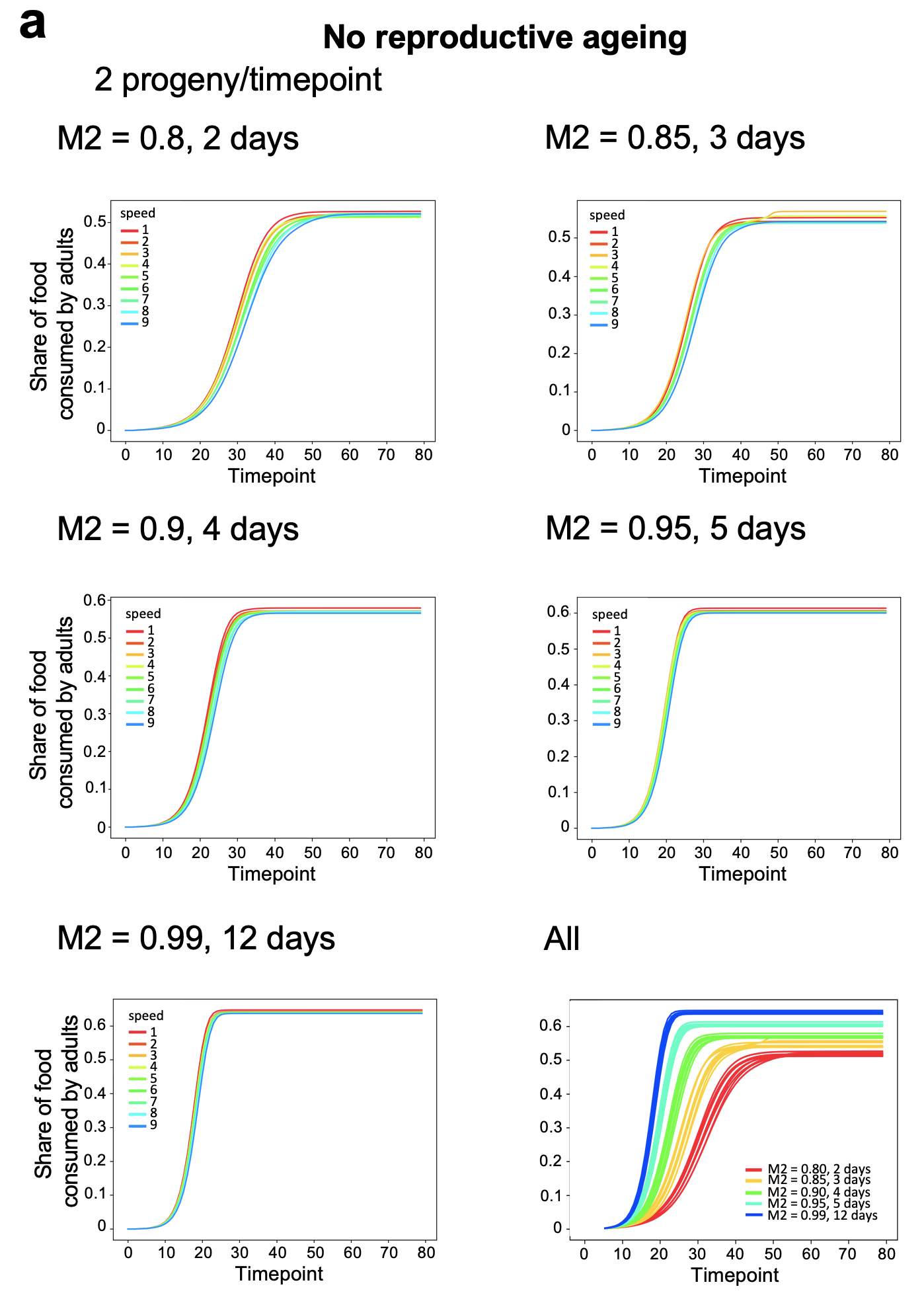
**

**
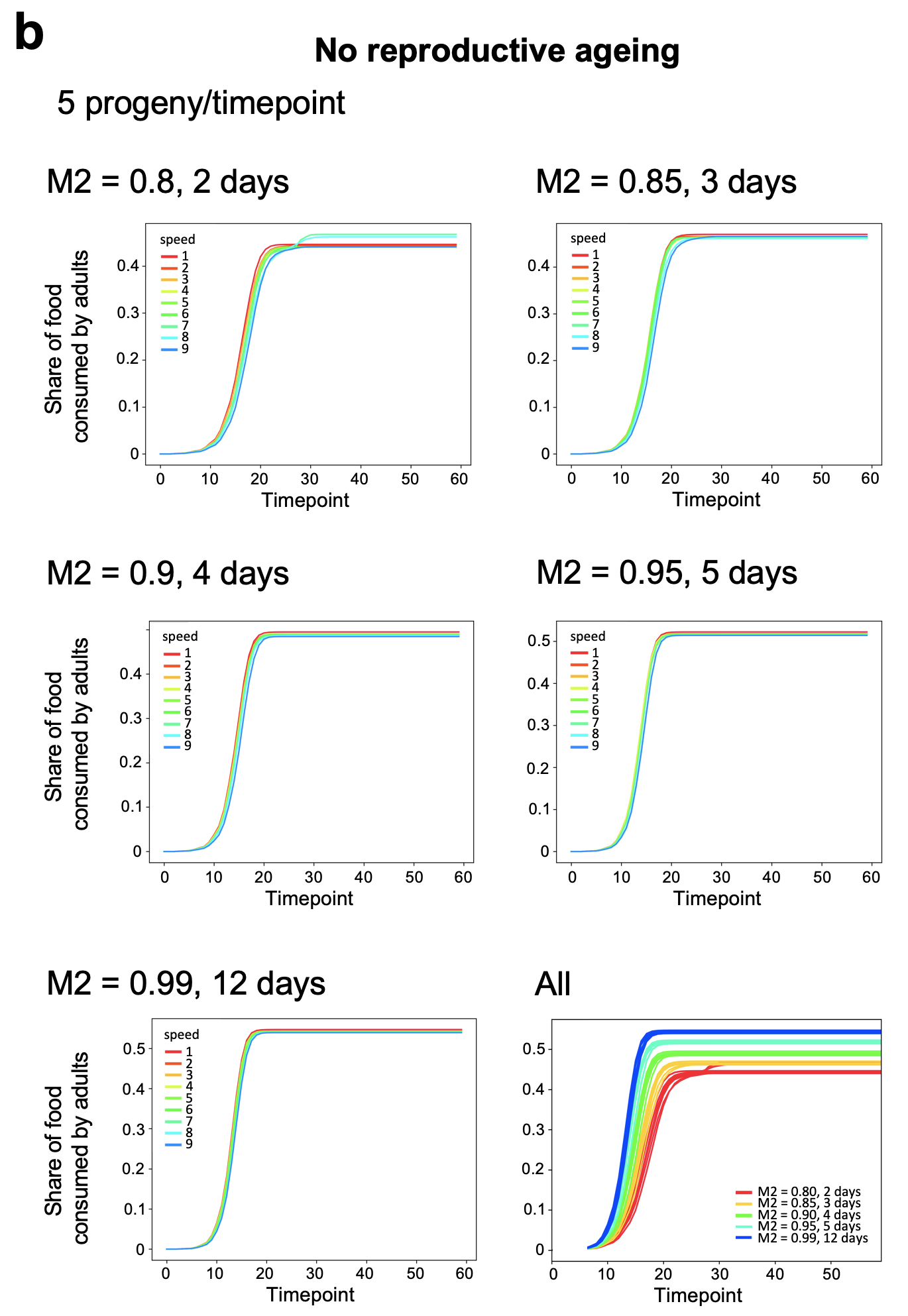
**

**
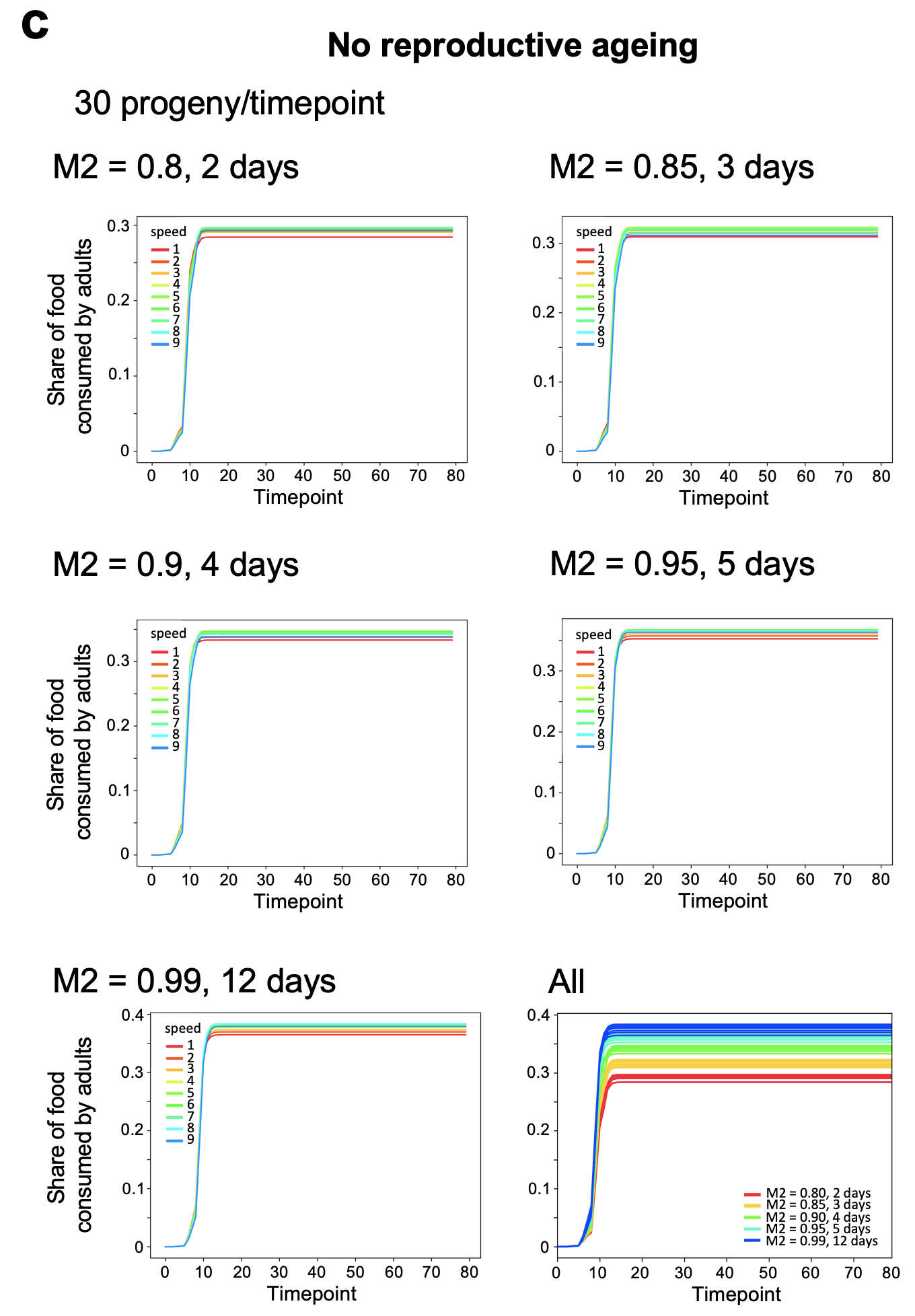
**

**
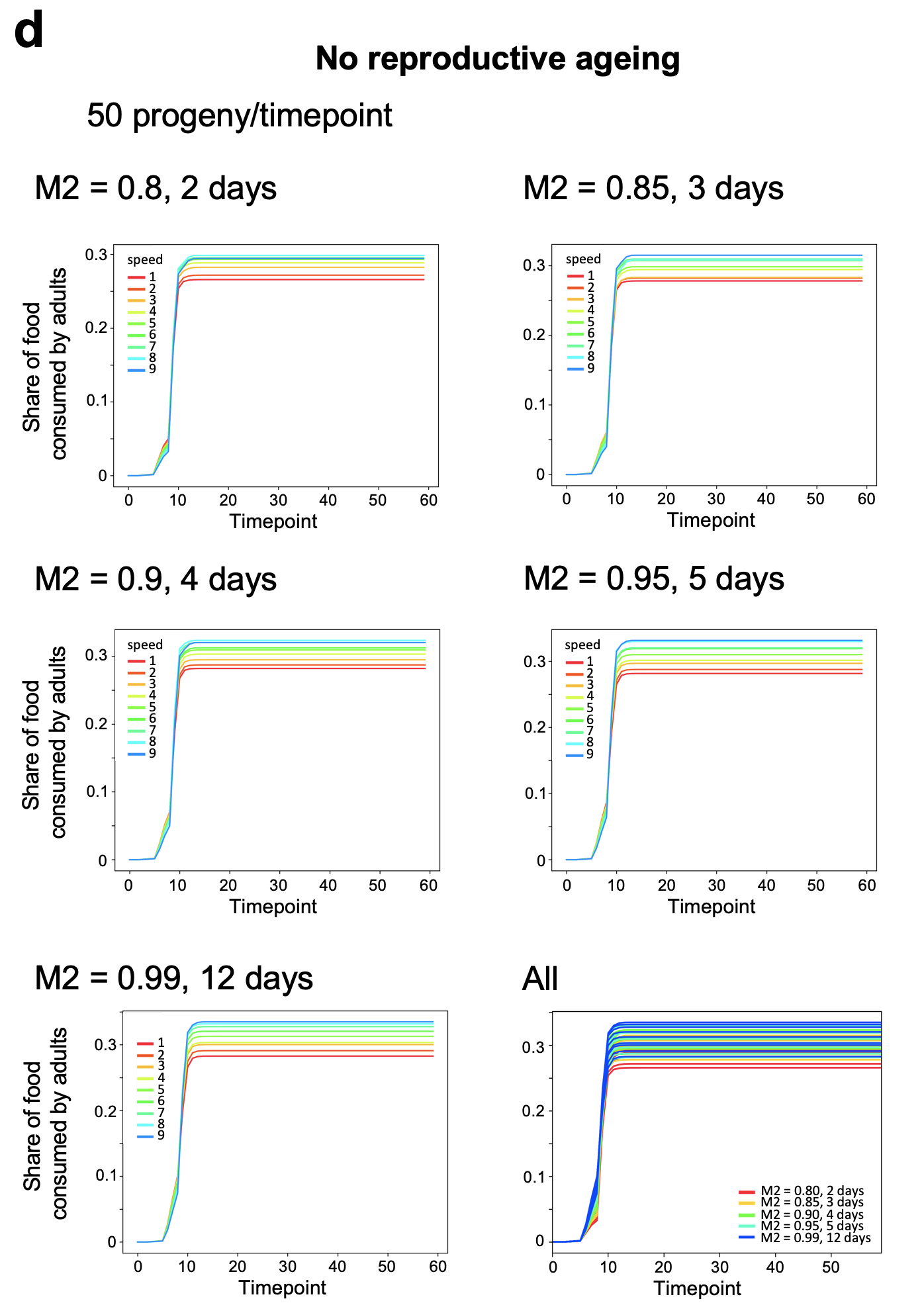
**

**Figure S3**  **Adult food consumption share when there is no reproductive decline with age.** For individual lifespan, is colour coding is for dispersal speed; for combined data (bottom right), colour coding is for lifespan. **a,** 2 progeny per timepoint , **b,** 5 progeny per timepoint, **c,** 30 progeny per timepoint, **d,** 50 progeny per timepoint.


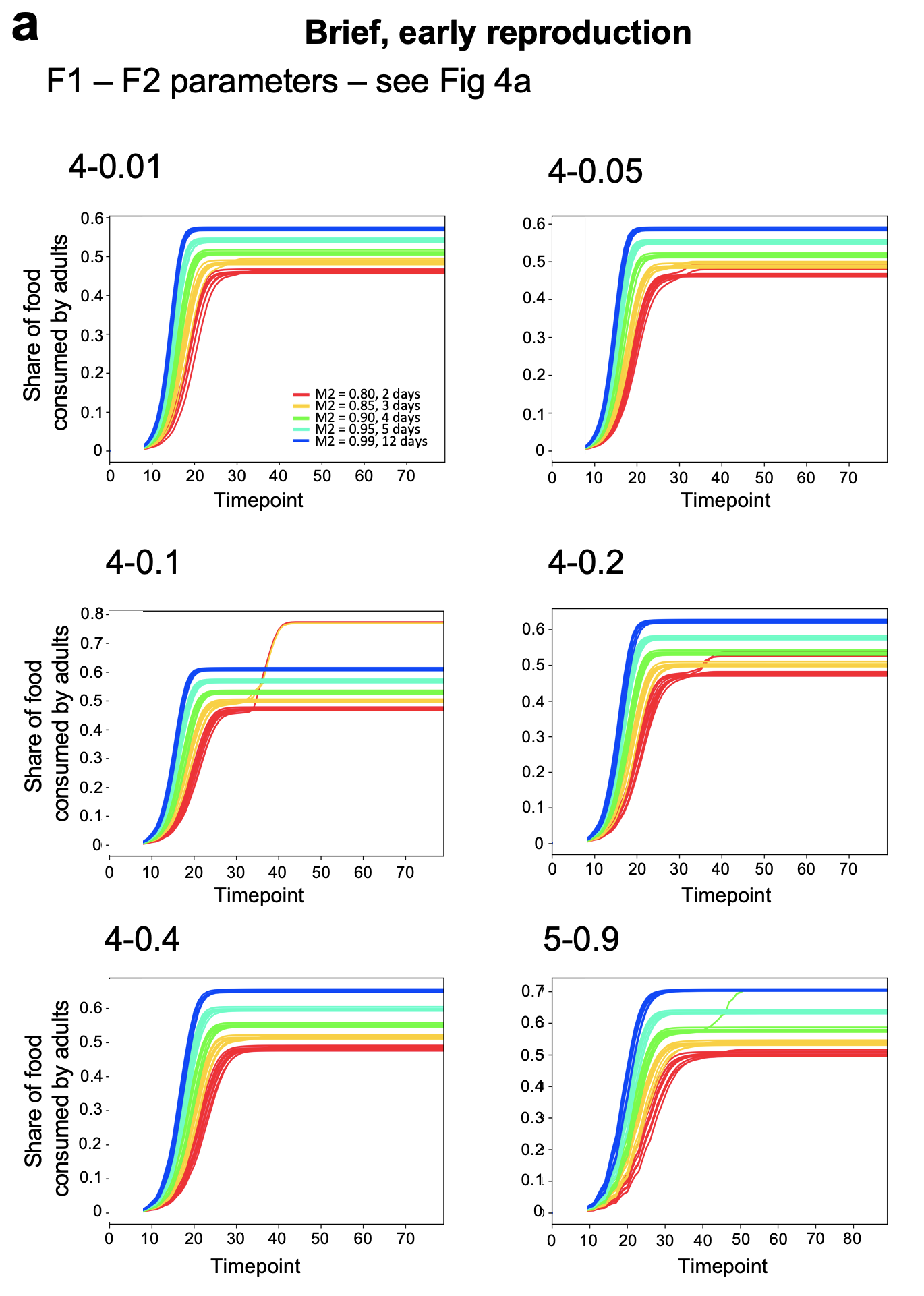


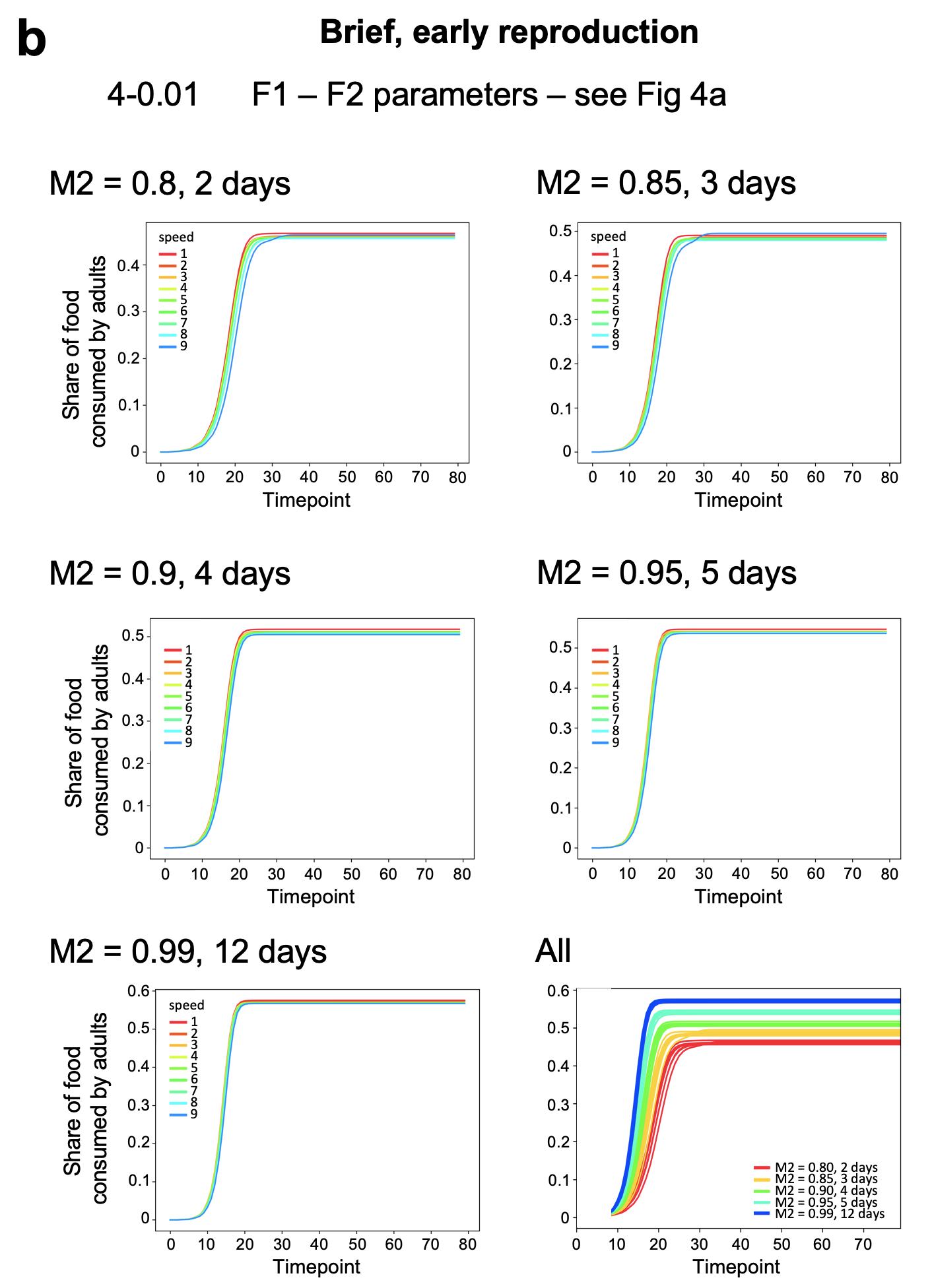


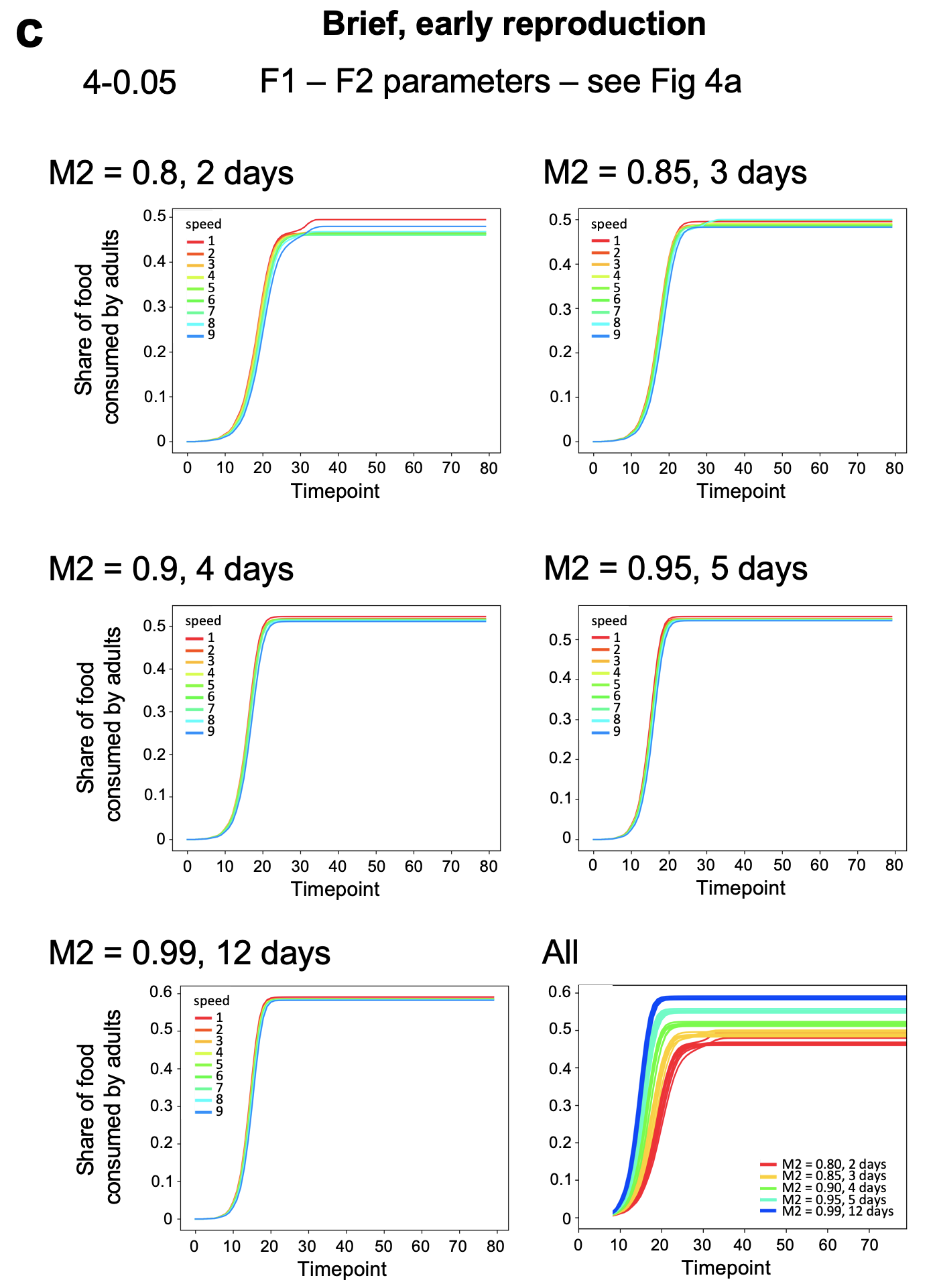


**
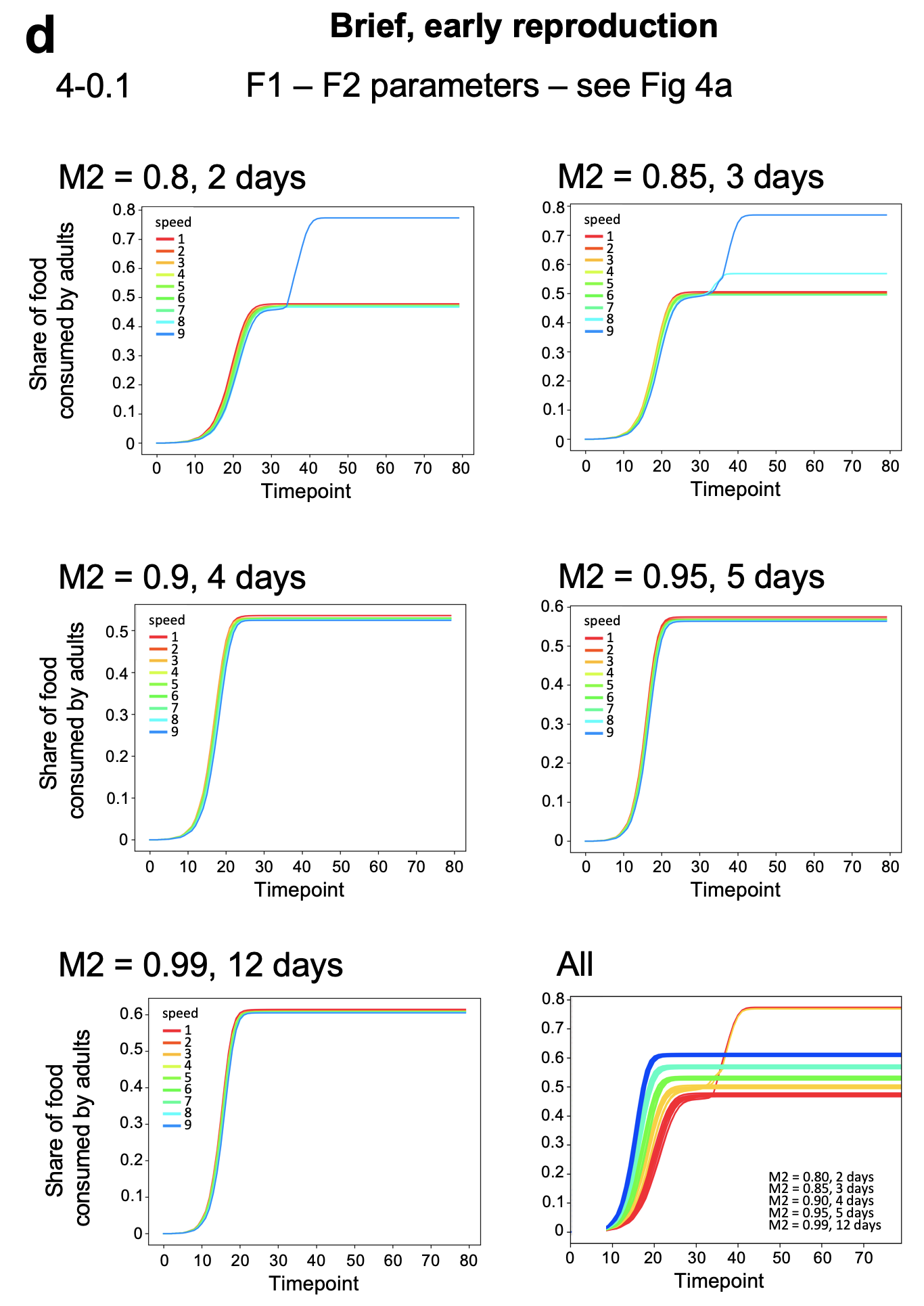
**

**
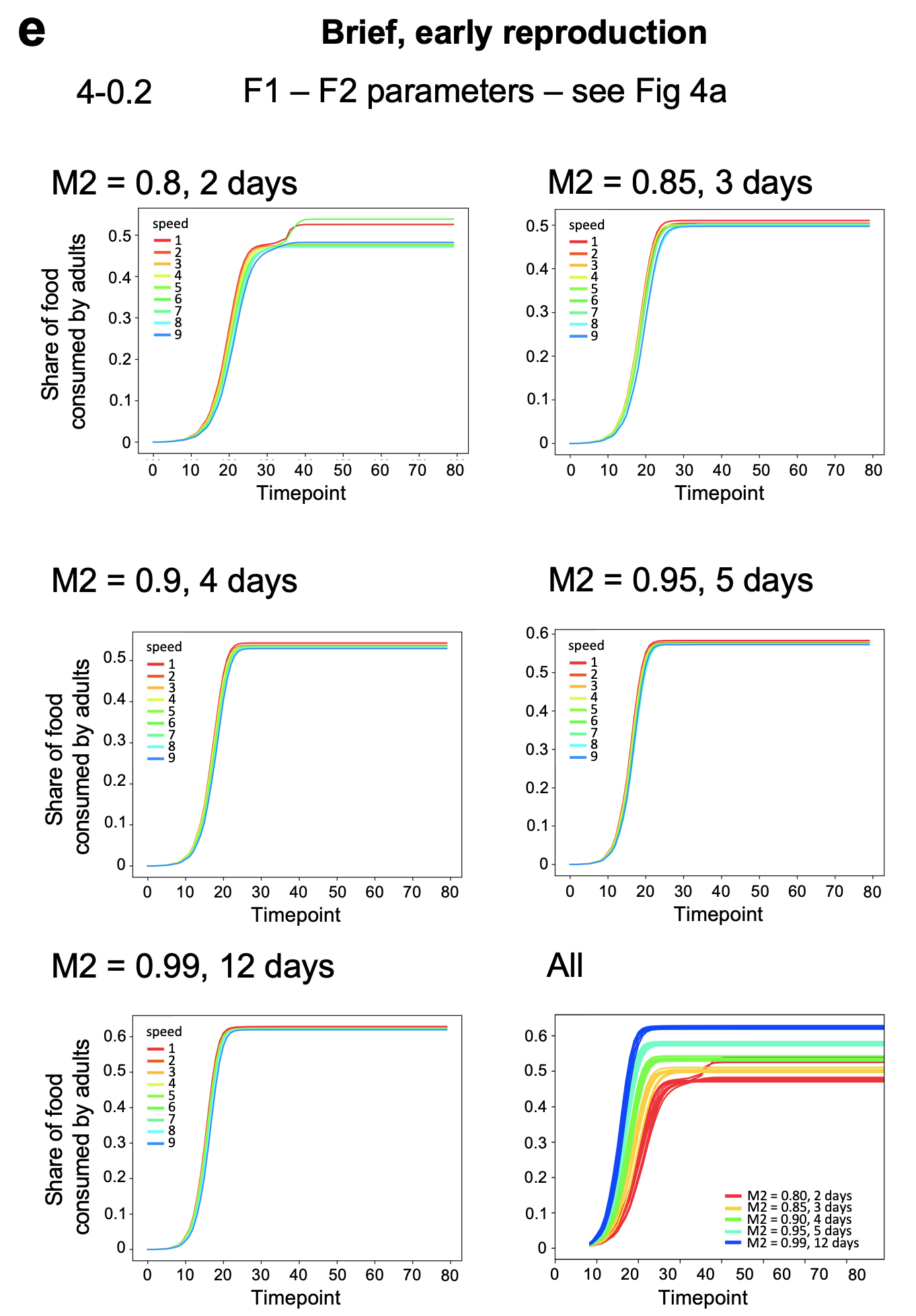
**

**
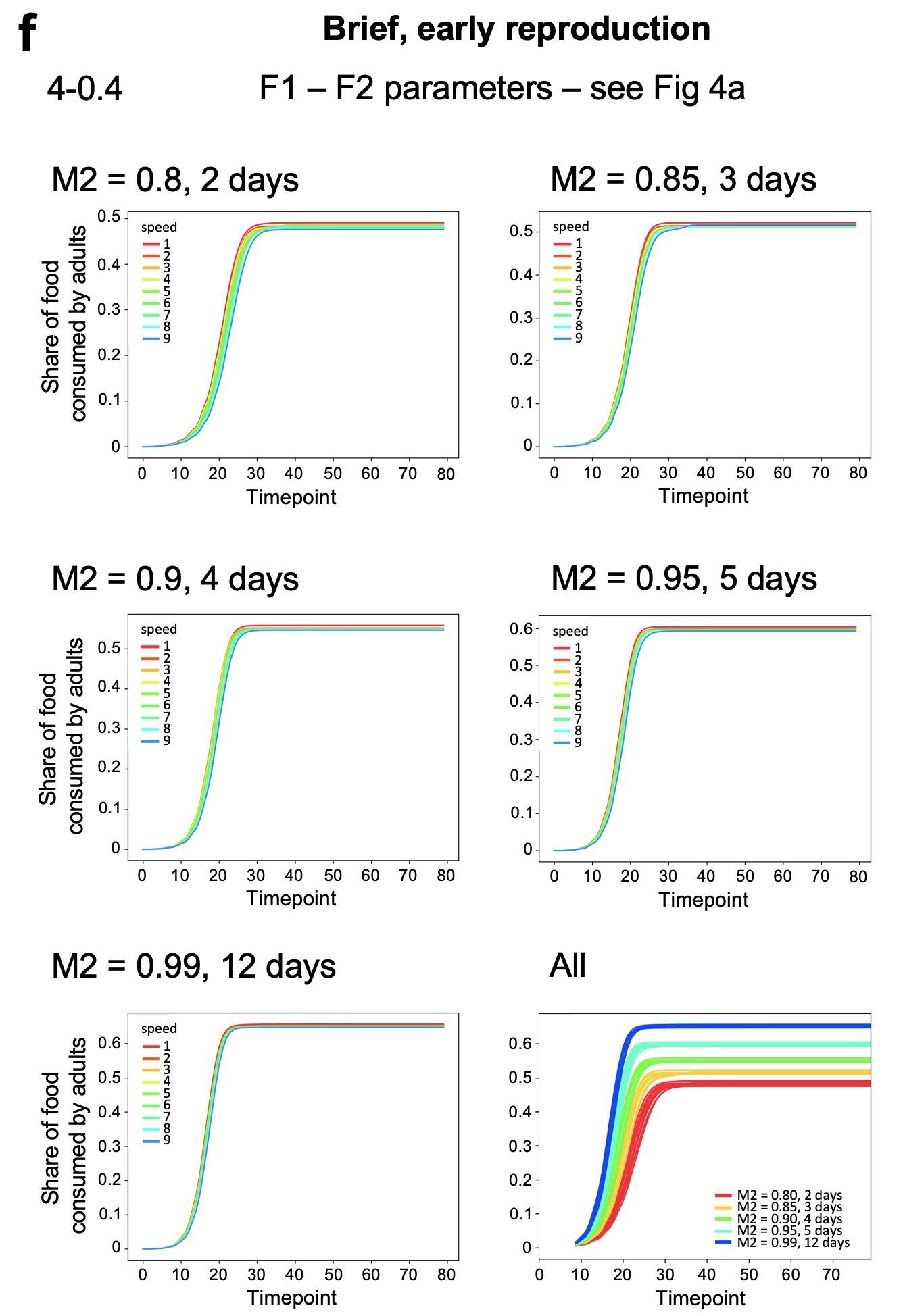
**

**
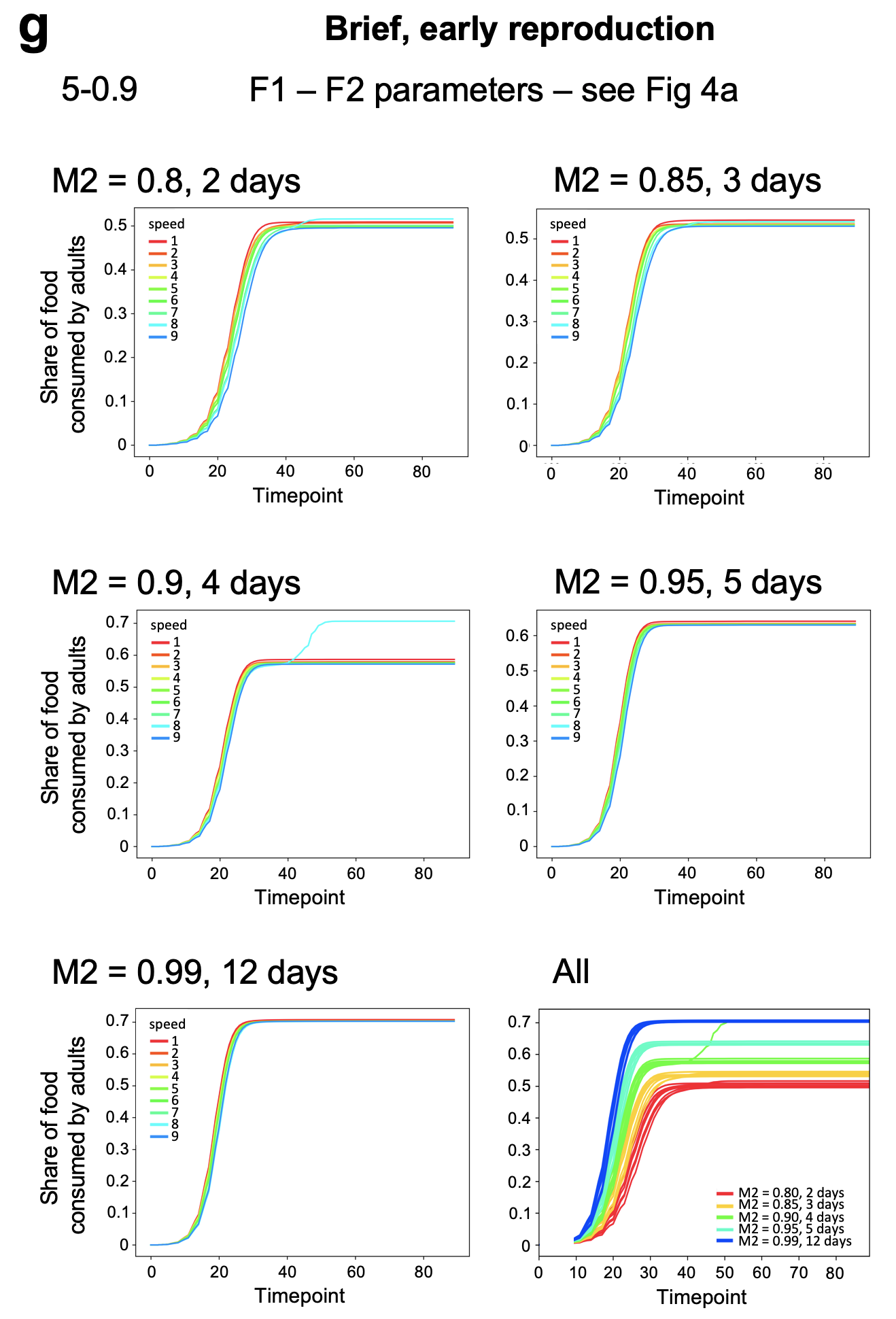
Figure S4 Adult food consumption share for different age-dependent reproductive spans.** For individual lifespan, colour coding is for dispersal speed; when all curves are combined (bottom right), colour coding is for lifespan. **a,** Different lifespans are combined, all various reproductive spans are shown together, **b,** F1-F2 parameters: 4-0.01, **c,** F1-F2 parameters: 4-0.05, **d,** F1-F2 parameters: 4-0.1, **e,** F1-F2 parameters: 4-0.2, **f,** F1-F2 parameters: 4-0.4, **g,** F1-F2 parameters: 5-0.9.

**
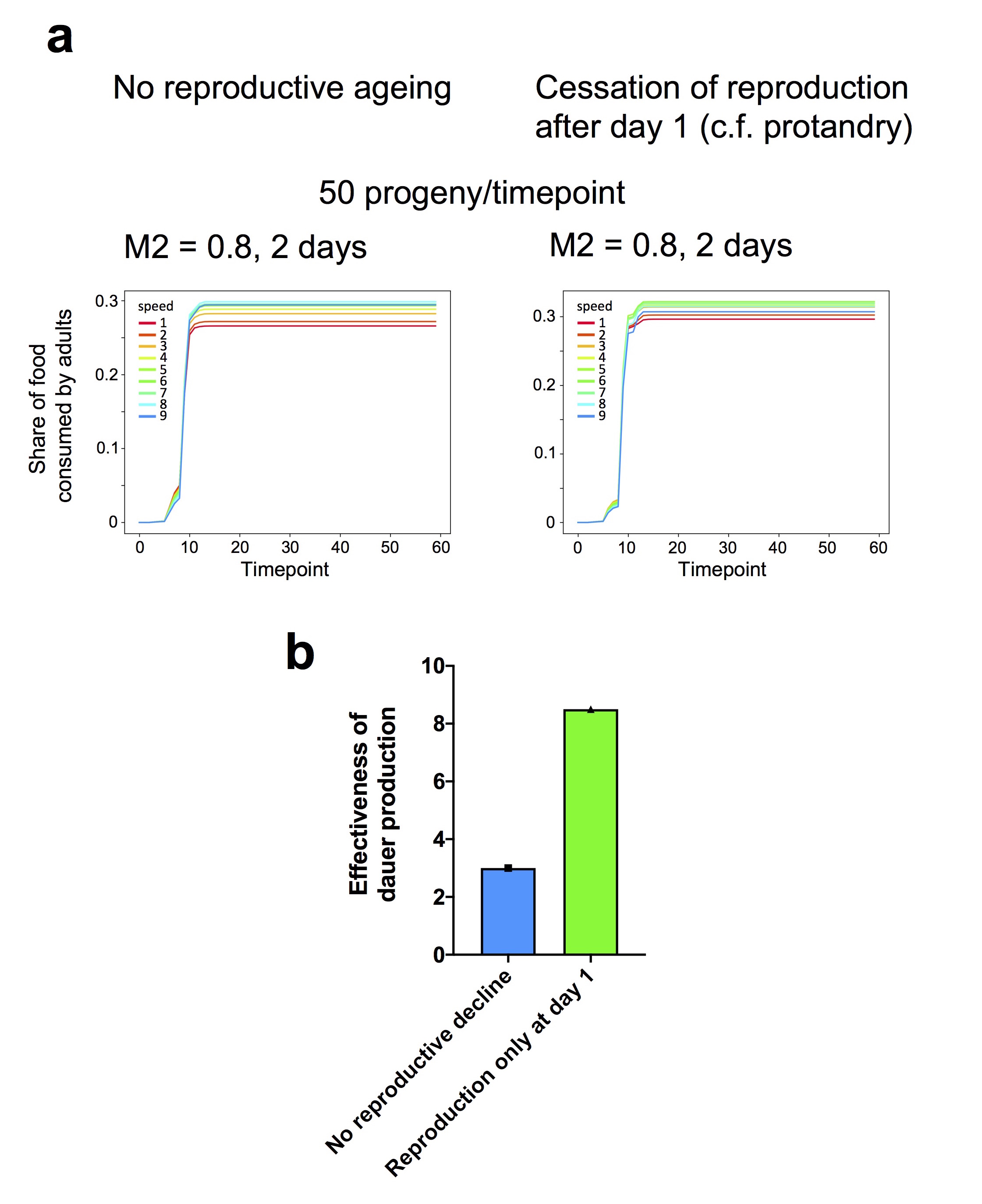
**

**Figure S5** **Reproduction on day 1 only increases fitness more than sustained reproduction when reproduction rate is high**. The data are shown for the shortest lifespan and highest viscosity when production rate is 50. **a**, Comparison of adult food consumption share for reproduction on day 1 only and sustained reproduction; colour coding is for different dispersal speeds. **b**, Efficiency of dauer production (sum of dauers/number of animals produced) for reproduction on day 1 only or with sustained reproduction.


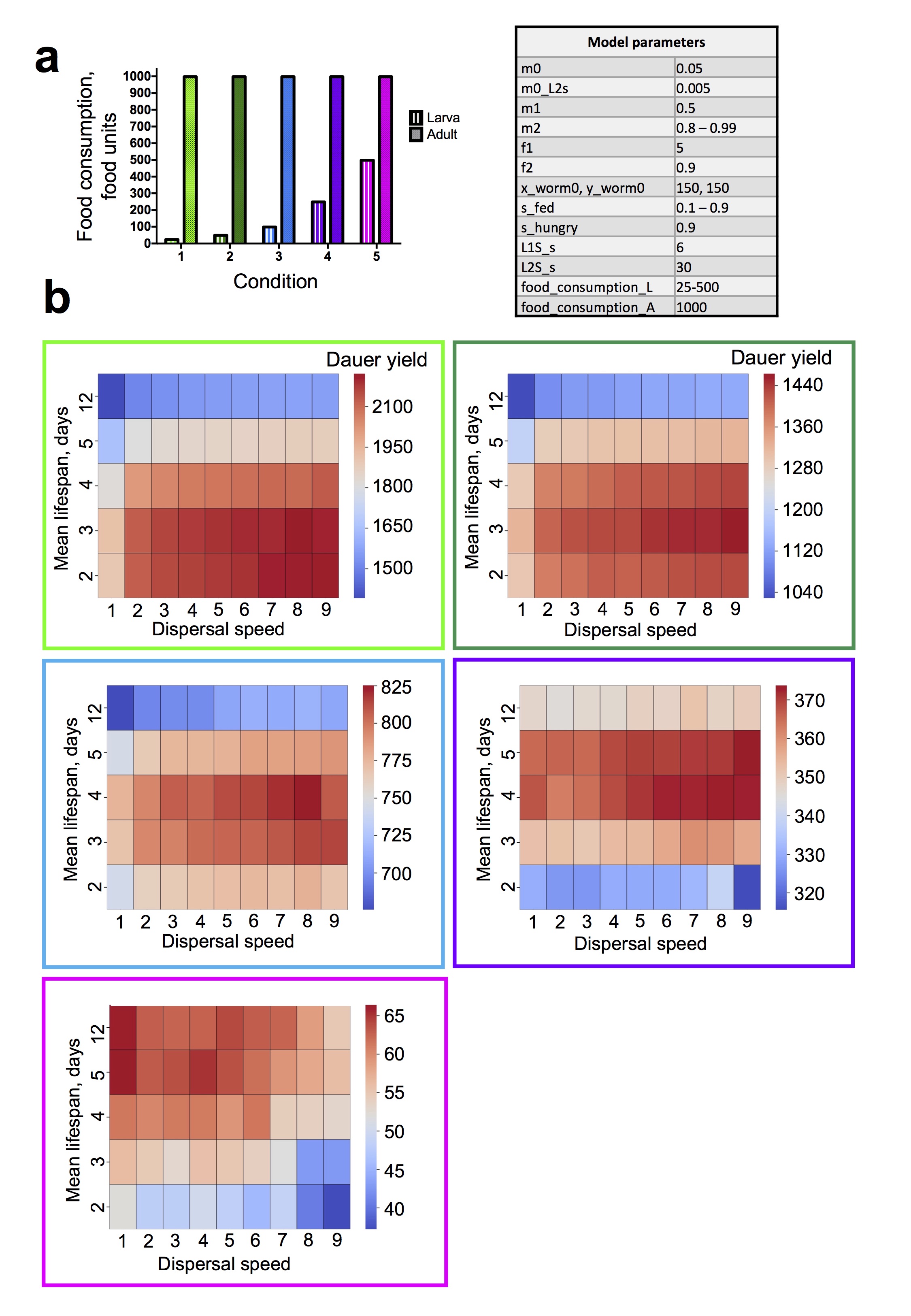


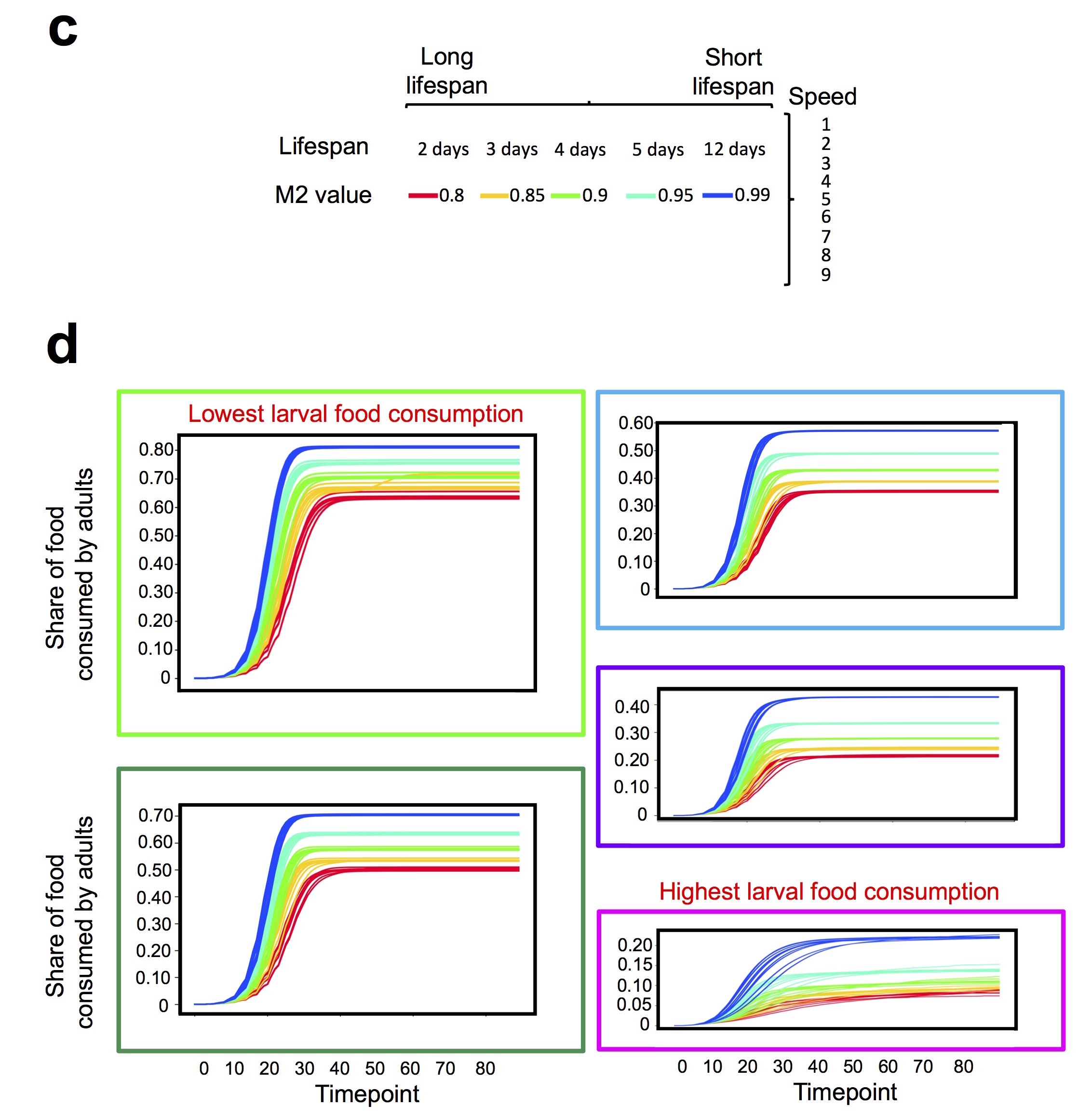


**
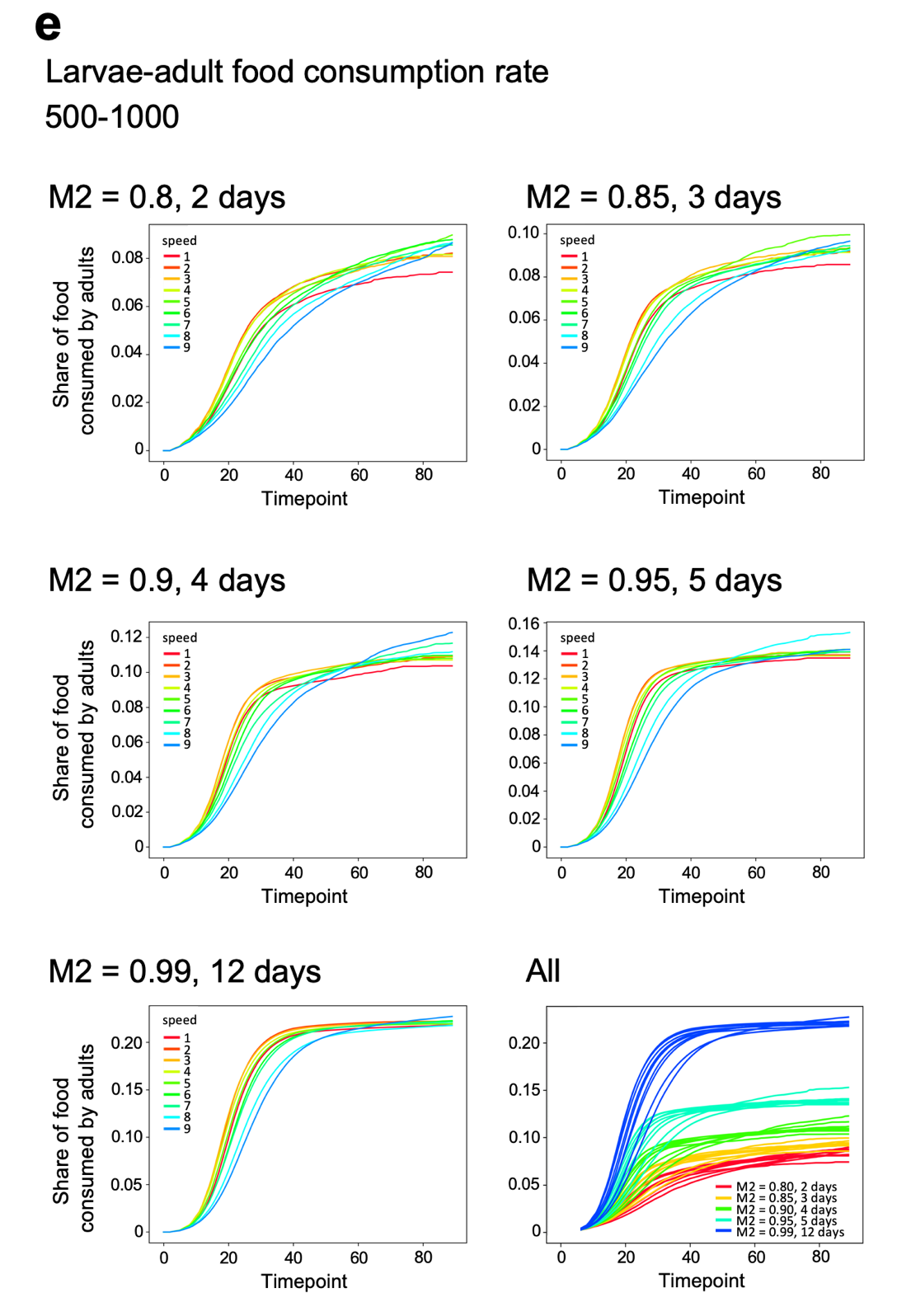
**

**Figure S6 Higher relative larval food consumption reduces the adaptive value of short lifespan**. **a**, 5 relative food consumption settings used in simulations. Colours used here indicate relative food consumption in plots in **b**, **d**. Reproductive schedule in all cases: 4 progeny on day 1 only. **b**, Colony fitness (dauer yield) given food consumption settings shown in **a**. Note that early death increases fitness when adults eat relatively more. **c**, **d** Share of food consumed by adults given different relative food consumption. **c**, Colour code for lifespan, various dispersal speed are marked with the same colour for each lifespan. **d**, Share of food consumed. **e,** Adult food consumption share when larvae consumption rate is only twice lower than in adults (marked with violet in **a**). **e,** Adult food consumption share when larvae and adult food consumption rates are 500 and 1000 food units, respectively. **d**, **e** For individual lifespan, dispersal speed is colour coded; when all curves are combined (bottom right), lifespan is colour coded.


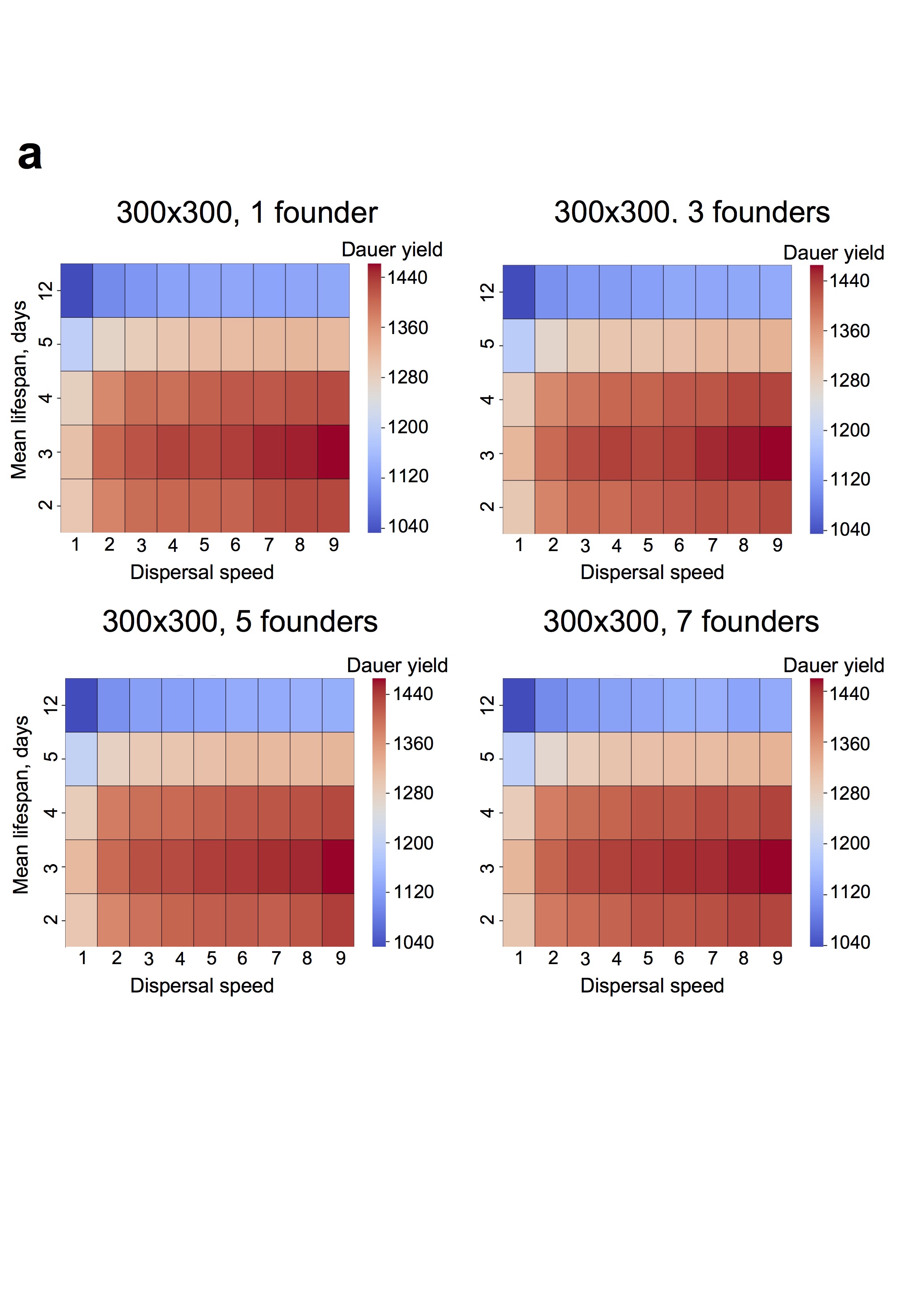


**
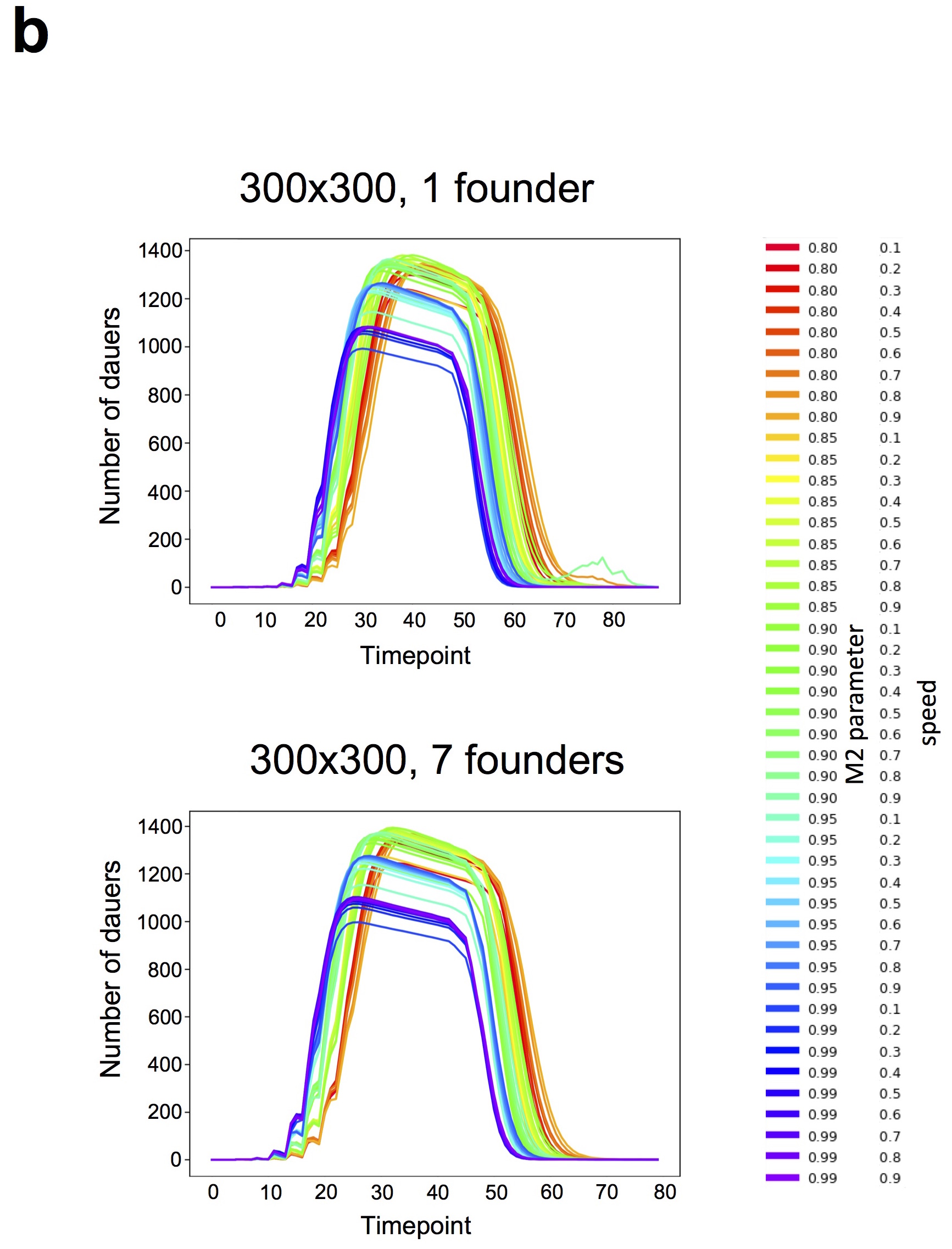
**

**Figure S7 Fitness benefit of early death is not sensitive to founder worm number**. **a**, Effects of lifespan and population viscosity on fitness (dauer yield) for different numbers of founders. Reproductive schedule in all cases: 4 progeny on day 1 only (other conditions are the same as in Figure 4). **b**, Number of dauers at a timepoint for 45 combinations of lifespans and dispersal speeds for 1 and 7 founders. Colour codes for various lifespans and dispersal speeds are described. The absence of an effect of founder number on dauer yield (**a**) is unexpected, and remains to be explained; however dauer number does increase faster when 7 founders establish a colony (**b**).

**
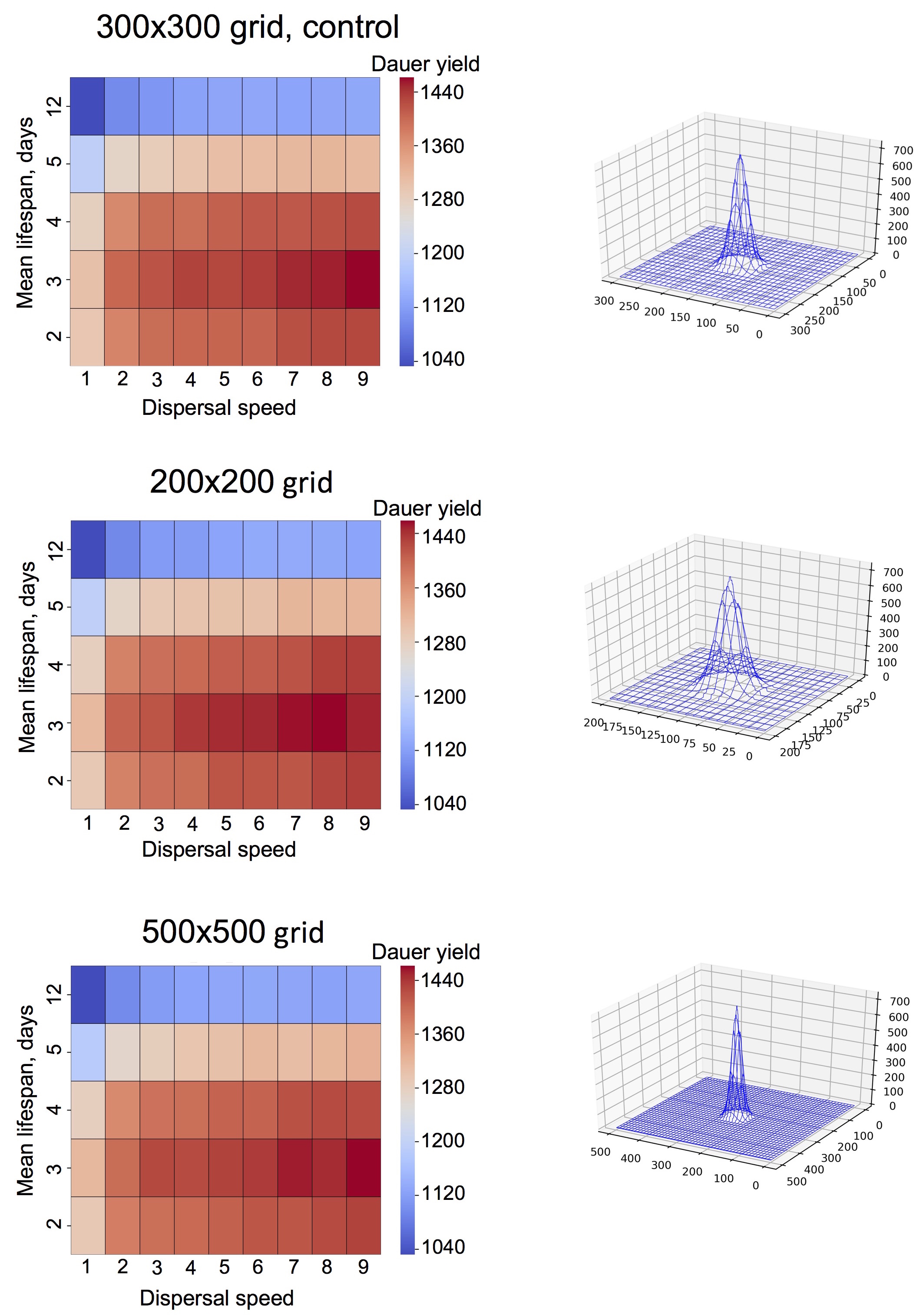
**

**Figure S8 Fitness benefit of early death is not sensitive to grid size**. Effects of lifespan and population viscosity on fitness (dauer yield) for different sizes of the grid. Reproductive schedule in all cases: 4 progeny on day 1 only (other conditions are the same as for Figure 4). Note that the optimal dispersal speed appears slightly lower for the smallest (200x200) grid.

**
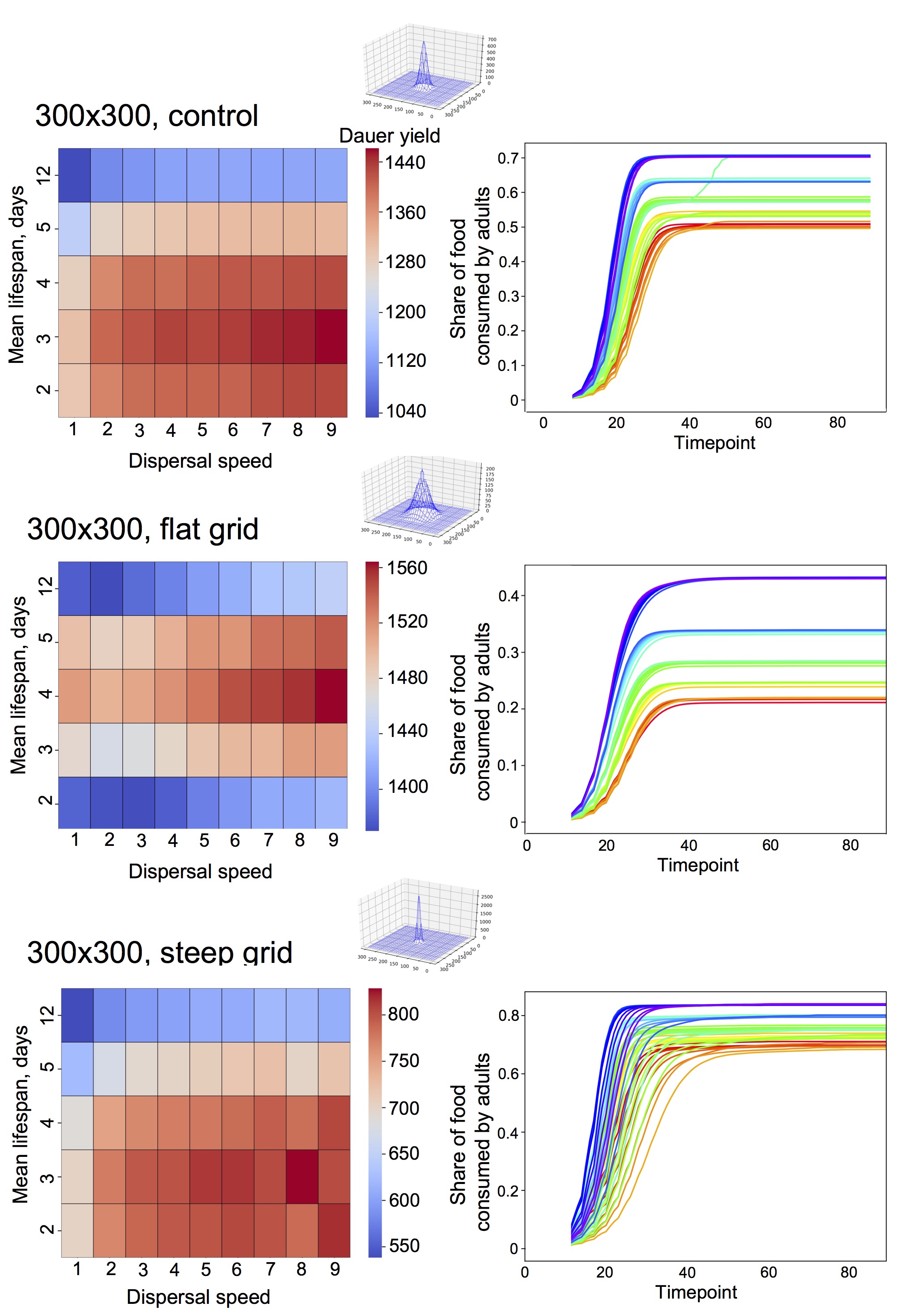
**

**Figure S9 Little effect of food patch morphology on optimal lifespan**. **Left column**, Effects of lifespan and population viscosity on fitness (dauer yield) for different shapes of the food source (indicated on the figure). Reproductive schedule in all cases: 4 progeny on day 1 only (other conditions are the same as for Figure 4). **Right column**, Share of food consumed by adults given different morphologies of the food source. For colour codes for the various lifespans and dispersal speeds, see Figure S7b. Note that a steeper food patch morphology decreases dauer yield, while a flatter one increases it. A possible explanation here is that adults consume food more slowly on a flatter food source, thereby allowing more larvae to accumulate.

**
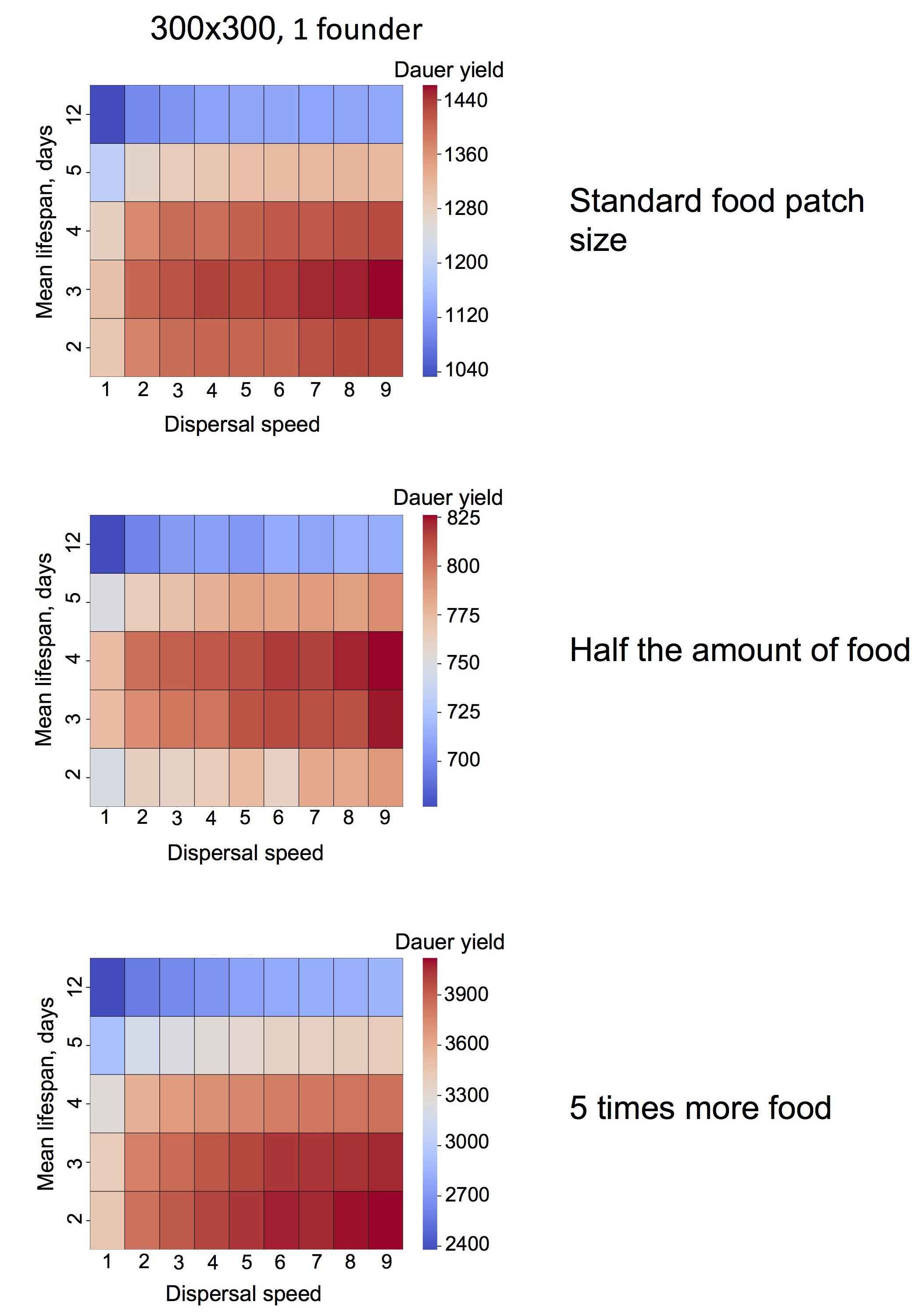
**

**Figure S10 Death is more beneficial when there is more food**. Effects of lifespan and population viscosity on fitness (dauer yield) given different food patch sizes. Reproductive schedule in all cases: 4 progeny on day 1 only (other conditions are the same as for Figure 4; adult food consumption rate is 1000).


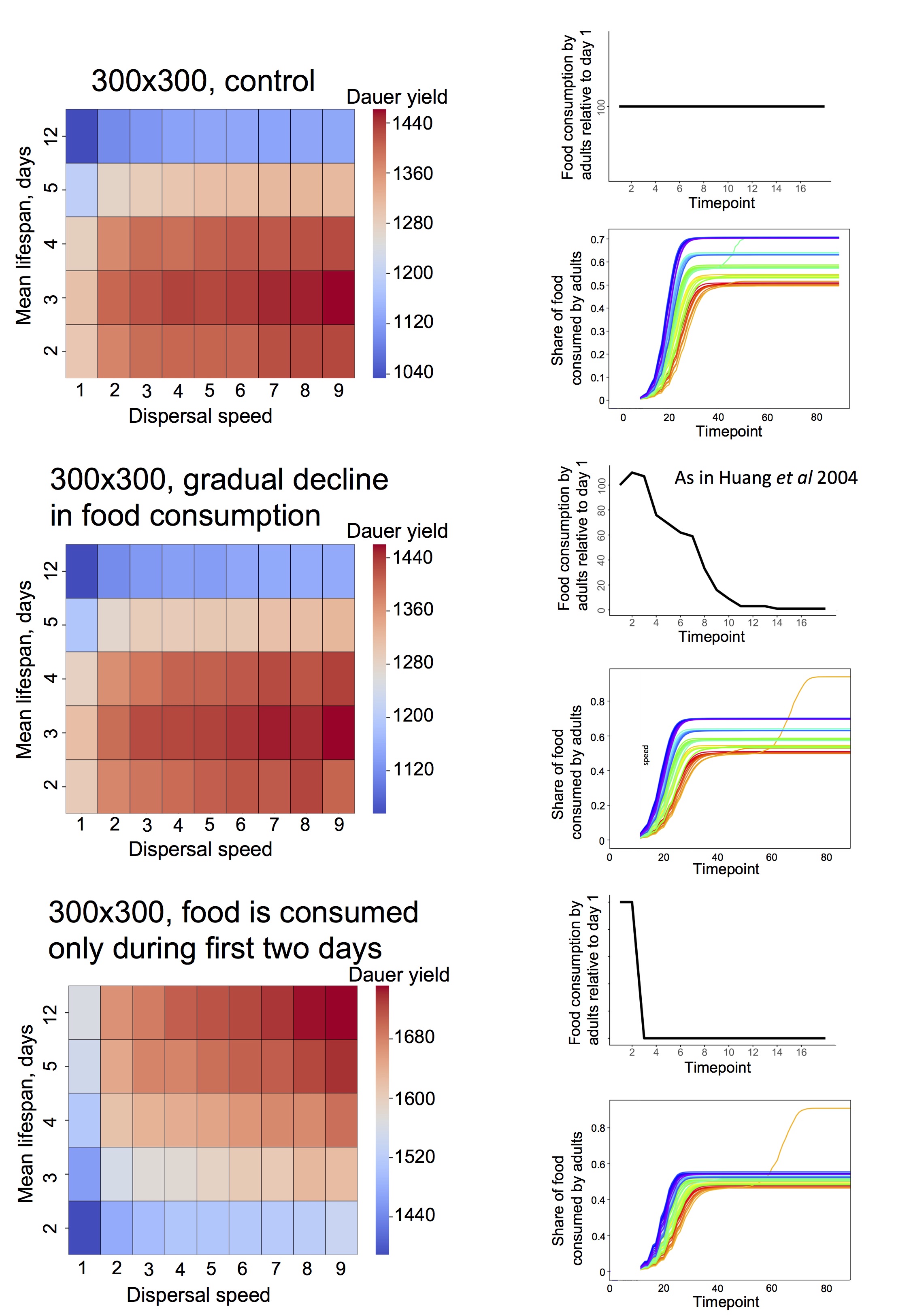


**Figure S11 The decline in food consumption by adults diminishes the value of adaptive death**. **Left column**, Colony fitness (dauer yield) for different adult food consumption schedules (indicated on the figure). Reproductive schedule in all cases: 4 progeny on day 1 only (other conditions are the same as for Figure 4). **Right column**, adult food consumption schedules and share of food consumed by adults. Colour codes for various lifespans and dispersal speeds are described on the Figure S7b.

**
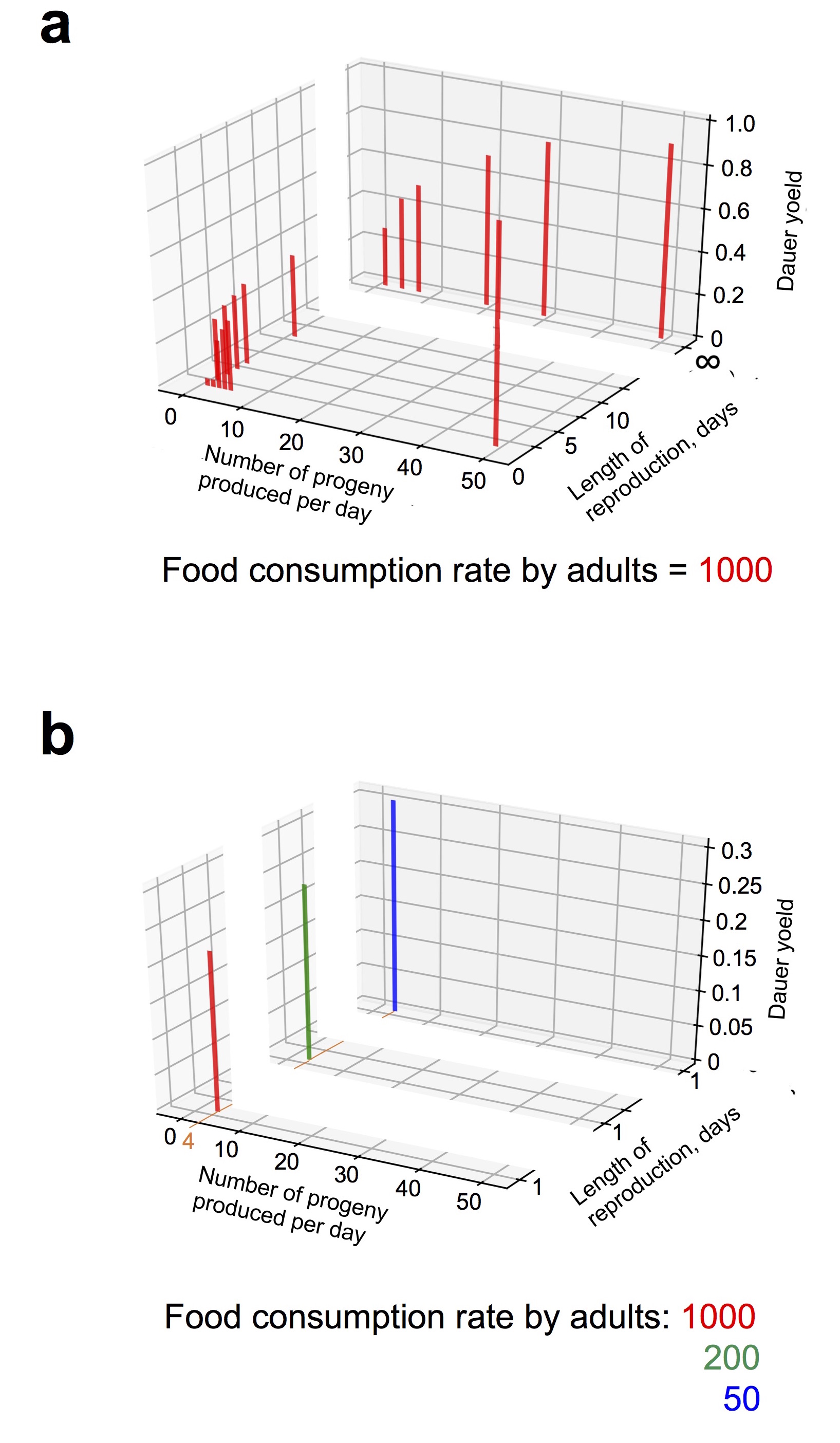
**

**Figure S12 Comparison of maximum dauer yield given a variety of conditions**. **a**, Maximum dauer yield among 45 ageing rate x dispersal speed combinations for various progeny production rates and durations of reproduction expressed relative to the highest one, which is 50 progeny only on day 1 (maximum dauer yield is 6400 dauers). Food consumption rate by adults was fixed at 1000. **b,** Relative maximum dauer yield among 45 ageing rate x dispersal speed combinations for various adult food consumption rates (indicated by colours). Reproductive schedule: 4 progeny on day 1 only (other conditions are the same as for Figure 4).


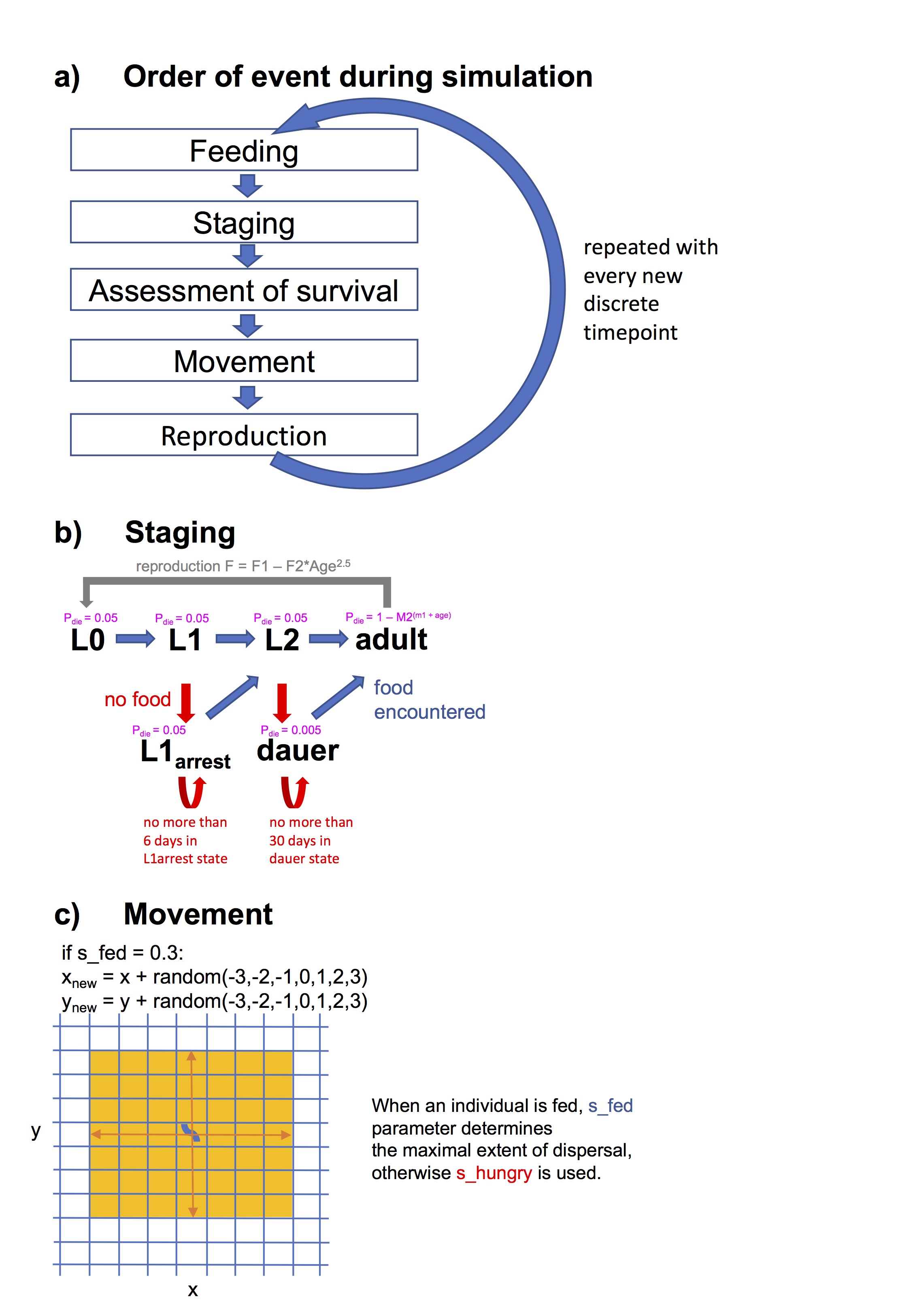


**Figure S13 A more detailed explanation of the model**. **a**, The scheme shows at what order each individual performs actions (feeds, then changes stage or ages, then survives, then moves, then reproduces) at each discrete timepoint. **b,** A scheme showing how an individual changes stage depending on fed status. It also shows formulas for calculation the probability of mortality and the number of progeny. **c,** Scheme illustrating how parameters s_fed and s_hungry limit maximal dispersal for an individual determining the area of possible translocation.
